# *CFTR* Gene Regulation in Human Pancreatic Duct, Bile Duct and Sweat Gland Epithelial Cells

**DOI:** 10.1111/jcmm.70751

**Published:** 2025-08-10

**Authors:** Ayushi Umrigar, Katreya Lovrenert, Jenny L. Kerschner, Neeraj Sharma, Britney Tian, Rebecca Melton, Kyu Shik Mun, Nirbhayaditya Vaghela, Mina Ogawa, Gedge D. Rosson, Shinichiro Ogawa, Anjaparavanda P. Naren, Kyle J. Gaulton, Shih‐Hsing Leir, Ann Harris

**Affiliations:** ^1^ Department of Genetics and Genome Sciences Case Western Reserve University School of Medicine Cleveland Ohio USA; ^2^ McKusick‐Nathans Department of Genetic Medicine The Johns Hopkins School of Medicine Baltimore Maryland USA; ^3^ McEwen Stem Cell Institute, Ajmera Transplant Centre Toronto General Hospital Toronto Ontario Canada; ^4^ Biomedical Sciences Program University of California San Diego La Jolla California USA; ^5^ Division of Pulmonary and Critical Care Medicine, Department of Medicine Cedars‐Sinai Medical Center Los Angeles California USA; ^6^ Division of Gastroenterology and Hepatology, Department of Medicine Cedars‐Sinai Medical Center Los Angeles California USA; ^7^ Department of Pediatrics University of California San Diego La Jolla California USA

**Keywords:** ATAC‐seq/snATAC‐seq, bile duct/cholangiocyte, cis‐regulatory elements, cystic fibrosis transmembrane conductance regulator gene, pancreatic duct, sweat gland

## Abstract

Epithelial cells at many sites in the body are affected by the inherited disorder cystic fibrosis (CF). The lung was the major focus of research until recently, when effective therapeutics became available for most people with CF. There is now renewed interest in CF aetiology in other locations in the body, where the regulatory mechanisms for the CF transmembrane conductance regulator (*CFTR*) gene are less well‐characterised. The definition of the genomic elements controlling *CFTR* expression and their associated transcription factors is important for the design of gene‐based therapies. Here we identify the *cis*‐regulatory elements (CREs) associated with the *CFTR* locus by open chromatin mapping in pancreatic adenocarcinoma cell lines, primary human pancreatic and bile duct (cholangiocyte) organoids and single cells from tissues, as well as sweat gland coil and duct epithelial cells. We show that broadly these cell types use a combination of CREs that were characterised previously either in airway or intestinal epithelial cells, though not occurring together in these two cell lineages. Moreover, the chromatin structure of the *CFTR* locus in pancreatic cell lines is consistent with earlier models. We also use bioinformatic tools to predict the transcription factor network in these rare cell lineages from open chromatin peaks genome‐wide.

## 1 Introduction

Specialised epithelial cell types in the lung are a major focus of research into the inherited disorder cystic fibrosis (CF). These cells are the primary targets for therapeutic gene replacement or gene editing, particularly for those people with CF (pwCF) carrying mutations that do not respond to current highly efficient modulator therapy (HEMT) (reviewed in [1, 2]). While lung disease underlies the life‐limiting aspects of untreated CF, many other organs are also affected, particularly with increasing longevity among pwCF. The cystic fibrosis transmembrane conductance regulator (*CFTR*) gene, errors in which cause CF, is expressed in ductal epithelial cells at many sites [3]. In the digestive system, the first evidence of CF pathology is before birth in the pancreas, where *CFTR* is highly expressed in the epithelial lining of the small ducts and in centroacinar cells [4]. The gene is also expressed in bile ducts of the liver/gall bladder [5] in addition to well‐studied regions of the small intestine and colon. *CFTR* transcripts are also abundant in sweat gland ducts [6] (at lower levels in the coil), consistent with the diagnostic elevated sweat chloride levels in pwCF, and in the collecting ducts of the kidney [3]. CFTR may have functions in other anatomical sites such as the brain and the heart, though its contribution to CF‐associated pathology in these organs in humans is less well‐characterised.

The regulatory mechanisms coordinating cell‐type‐selective expression of *CFTR* are best characterised in secretory epithelial cells of the airway and intestinal epithelium (reviewed in [7]). The *CFTR* gene promoter apparently lacks cell‐specific control elements, so extensive efforts to identify *cis*‐regulatory elements (CREs) for the gene, both in cell lines generated from these sites and in primary cell cultures, were necessary to provide a robust knowledge base. To summarise, cell‐selective transcription factor‐dependent CREs are recruited to the *CFTR* gene promoter by looping mechanisms, which are associated with changes in higher‐order chromatin structure at the locus. Structural elements at the topologically associating domain (TAD) boundaries of the locus (−80.1 and +48.9 kb from the promoter and 3′UTR, respectively) are apparently utilised in all cell types irrespective of *CFTR* expression, as are a number of critical sites of occupancy of CCCTC‐binding factor (CTCF) and cohesin complex at −20.9 kb and within several introns of the gene [8, 9]. In primary human bronchial epithelial (HBE) cells and many airway epithelial cell lines, the key CREs are located outside the gene in regions within ~30 to 45 kb upstream of the basal promoter and downstream of the 3′ end of the gene [10]. These elements recruit both epithelial TFs, such as Krüppel‐like factor 5 (KLF5) [11] and Ets homologous factor (EHF) [12], but also more general TFs that are involved in the immune response and oxidative stress, among others [13, 14]. In contrast, in intestinal organoids and colon cancer cell lines, the important CREs are found within introns of the gene where they work coordinately to drive gene expression [8, 15, 16]. The intestinal CREs bind TFs that commonly regulate genes in the digestive system, including hepatocyte nuclear factor 1 alpha and beta (HNF1α/β), HNF4 and caudal type homeobox 2 (CDX2) [17], among many others. Most recently we examined *CFTR* regulation in primary human epididymis epithelial cells which contribute to the male infertility that is associated with CF. In these cells, in addition to observing CREs that are characteristic of both airway and intestinal epithelial cells, we found a new CRE at −9.5 kb with respect to the gene promoter [18]. This element binds the androgen receptor, and though it is likely not a classical enhancer since its activation alone does not augment CFTR expression, co‐activation of the gene promoter facilitates its enhancer activity.

Here we focus on *CFTR* expression in cell types that are less well‐characterised for gene regulatory mechanisms and yet pivotal in CF pathology, including pancreatic duct cell lines, primary human pancreatic duct cell organoids [19], bile duct epithelial cell (cholangiocyte) organoids [20] and sweat gland duct and coil cells [21]. Our goal was to determine whether the CREs found in airway and intestinal epithelial cells are utilised in these other cell types, either individually or in combination, or whether additional cell types have novel regulatory landscapes. We find that the pancreatic duct cell lines exhibit open chromatin peaks at a combination of CREs that are already well defined in intestinal and airway epithelial cells but are not shared by these different cell types. Fewer peaks of open chromatin are seen in organoids derived from primary pancreatic duct cells and cholangiocytes, suggesting either a subset of epithelial cell types populate the organoids, a more uniform differentiation occurs under organoid growth conditions and/or cancer‐associated changes are a feature of the cell lines. Primary human sweat gland coil and duct epithelial cells show a very similar pattern of open chromatin, which has some unique characteristics. However, though each cell type analysed here exhibits a slightly different open chromatin peak signature, all conform to the model of an invariant *CFTR* TAD within which a set of CREs cooperate to fine‐tune gene expression. We also use open chromatin data to predict TFs that regulate gene expression in each of these CF‐relevant primary cell types.

## 2 Materials and Methods

### 2.1 Cell Culture

Human pancreatic adenocarcinoma cell lines BxPC‐3 (ATCC CRL‐1687) [22], Capan‐1 (ATCC HTB75) [23] and NP31 [24] were grown as described by ATCC or in the original published protocols. All cell lines were monitored to confirm maintenance of the original morphology (ATCC images) with serial passage, and *CFTR* expression levels were assayed regularly. Pancreatic cell lines were harvested at confluence.

Human pancreas organoids [19] and hES‐derived cholangiocyte organoids [20] were generated and cultured as described in referenced protocols and used at low passage numbers. Human sweat gland duct and coil cultures were established and differentiated according to the published protocol for mixed duct and coil cultures [21] and used at p1.

### 2.2 ATAC‐Seq

Omni‐ATAC‐seq was performed on 50,000 cells as described previously [25]. Library size distributions were visualised by TapeStation (Agilent) and quantified using the KAPA Library Quantification Kit (Roche). Libraries were pooled and sequenced on NextSeq 550 or NovaSeq X platforms. Data were processed and peaks were called by the ENCODE‐DCC/atac‐seq‐pipeline with default parameters. Technical replicates were generated for all cell types, and results are presented as irreproducible discovery rate (IDR). The HOMER 2 v4.11 pipeline [26] was used for known and de novo motif finding within a 200 bp window centred on identified peaks using default parameters.

### 2.3 ChIP‐Seq and ChIP‐qPCR for Histone Modifications

Chromatin immunoprecipitation (ChIP), was performed by standard protocols for ChIP‐qPCR and ChIP‐seq [8, 27]. Antibodies were specific for H3K27Ac (Millipore 07‐360), H3K27me3 (Millipore 07‐449) and Rabbit IgG (Millipore 12‐370). Due to the known robustness and reproducibility of the antibody, H3K27Ac data are shown from a single experiment, though IDR peak calls from technical replicates are shown below each track. Raw reads were processed using the AQUAS Transcription Factor and Histone ChIP‐Seq processing pipeline (https://github.com/ENCODE‐DCC/chip‐seq‐pipeline2) using the ENCODE (phase‐3) guidelines on the hg19 reference genome. This includes mapping using BWA and peak calling with MACS2. For ChIP‐qPCR, primers are shown in Table S1.

All genome‐wide data are deposited at GEO: GSE283992.

### 2.4 4C‐Seq

4C‐seq libraries were generated from cultured cells as described previously [28]. For 4C experiments, NlaIII and Csp6I were used as the primary or secondary restriction enzymes, respectively. Enzyme pairs and primer sequences used to generate 4C‐seq libraries for each viewpoint are shown in Table S2. Primers containing a 2‐nucleotide barcode at the 3′ end of the P5 linker were used to enable multiplexing of libraries generated from the same viewpoint on the same flow cell for sequencing. The sequencing data were processed using the 4C‐seq pipe protocol [29]. All 4C‐seq images were generated using the default parameters of the pipeline.

### 2.5 Western Blots

Cells were lysed in NET buffer (10 mM Tris–HCl, pH 7.5, 150 mM NaCl, 5 mM EDTA, 1% (v/v) Triton X‐100, 1X Protease Inhibitor Cocktail (Sigma‐Aldrich, P8430)) as described previously [30] for CFTR protein and resolved by standard SDS‐PAGE protocols. 30 μg of total protein were loaded for each cell line. Membranes were probed with antibodies specific for CFTR (Cystic Fibrosis Foundation, Ab‐596), β‐tubulin (Sigma‐Aldrich, T4026) and anti‐mouse‐IgG‐HRP (Agilent/Dako, P0447) and proteins were detected with ECL Western Blotting Substrate (Pierce).

## 3 Results

### 3.1 *CFTR* Regulatory Elements in Pancreatic Duct Epithelial Cell Lines

**FIGURE 1:**
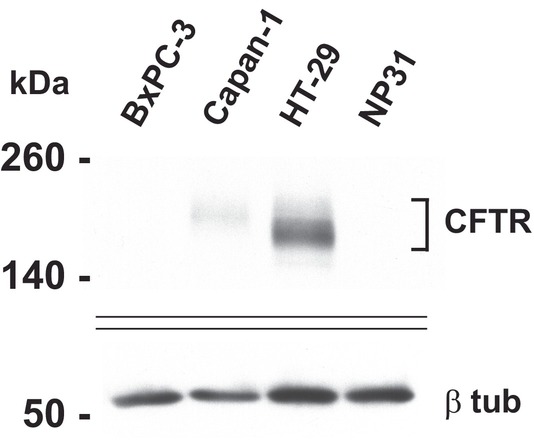
CFTR protein is expressed at a low level in Capan‐1 pancreatic adenocarcinoma cells but is absent from BxPC‐3 cells. The western blot shows protein extracted from BxPC‐3, Capan‐1, HT‐29 and NP31 cells. CFTR protein is detected with Ab596 compared to the β‐tubulin loading control.

Unlike the airway and intestinal epithelium, access to primary human pancreatic duct epithelial cells is limited, so we first examined open chromatin in pancreatic cancer cell lines to look for CREs. In earlier surveys of pancreatic adenocarcinoma cell lines by RT‐qPCR, we found CFTR mRNA only in Capan‐1 and NP31 cells and not in BxPC‐3 or seven other pancreatic adenocarcinoma cell lines [31, 32]. We validated the earlier RNA data by examining CFTR protein levels in Capan‐1, NP31 and BxPC‐3 cells by western blot, using the CFTR‐expressing colon carcinoma cell line HT29 [23] as a positive control (Figure 1). CFTR abundance in Capan‐1 cells was low compared to HT29 and at even lower levels in NP31 (only detectable on long ECL exposures) and was absent from BxPC‐3 cells. Next, we looked for *CFTR* CREs in the pancreatic cell lines by mapping open chromatin across the locus.

**FIGURE 2:**
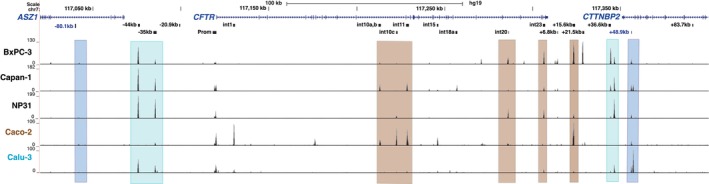
Open chromatin maps at the *CFTR* locus in pancreatic adenocarcinoma cells compared to intestinal (Caco‐2) and airway (Calu‐3) epithelial cells. Open chromatin maps generated by Omni‐ATAC‐seq of BxPC‐3, Capan‐1, NP31, Caco‐2 and Calu‐3 cell lines are shown. Each track shows merged data from 2 technical replicates. The locations of *ASZ1, CFTR* and *CTTNBP2* are shown at the top of the figure. Key DHS identified at the *CFTR* locus in all cells, and other cell‐selective DHS of interest are marked below the gene track in black. This manuscript uses legacy nomenclature for the *CFTR* gene to be consistent with our earlier work (see Table 1 for conversion to RefSeq). TAD boundaries are shown by blue boxes at −80.1 and +48.9 kb. Airway selective DHS at −35, −44 and +36.6 kb are shown by teal boxes. Intestinal selective DHS at intron 10a,b, intron 10c, intron 11, intron 20, intron 23 and +15.6 kb are shown by brown boxes.

Open chromatin profiles at the *CFTR* locus in BxPC‐3, Capan‐1 and NP31 cells were generated by ATAC‐seq (Figure 2) and compared to well‐characterised open chromatin maps in colon carcinoma (Caco‐2) and lung adenocarcinoma (Calu‐3) cells that we published earlier [16, 33]. Importantly, these two lines show the same peaks as primary human colon organoids and primary human bronchial epithelial cell‐selective CREs, respectively. Of note, all our previous data used legacy nomenclature for *CFTR* introns, so to avoid confusion we have also used this in the current work. However, to provide additional clarity, Table 1 shows both legacy and RefSeq nomenclature for each element. Moving from left to right across the *CFTR* locus in Figure 2, the first notable open chromatin peaks in the three pancreatic lines are the −44 and −35 kb sites (turquoise box) that are seen in Calu‐3 cells and are features of airway epithelial cells. In the CFTR‐negative BxPC‐3 line, the −35 kb site is just visible, unlike in the *CFTR*‐expressing Capan‐1 and NP31 lines, where the ratio of heights of the −35 and −44 kb sites are more similar. This observation is consistent with the known enhancer activity of the −35 kb CRE. Also highlighted by a turquoise box at the 3′ end of the locus are a pair of open chromatin peaks at ~+36.6 kb downstream of the last coding base in the *CFTR* transcript, which are evident in the three pancreatic lines but were previously defined as airway epithelial cell‐selective and are seen in Calu‐3 and HBE cells [33]. The ratio of heights of the two +36.6 kb peaks is different in the CFTR‐expressing pancreatic cells, with the distal peak higher than the proximal one in Capan‐1 and NP31, consistent with Calu‐3 below, and in contrast with BxPC‐3, where the proximal site is dominant to the distal one. These data suggest that the distal site may be associated with an enhancer element. Next, considering peaks of open chromatin marked in brown boxes on Figure 2, which denote CREs defined in intestinal epithelial cells, specifically introns 10 and 11 legacy (now introns 11 and 12, Table 1). BxPC‐3 cells have very little open chromatin across this region; NP31 cells have a minor peak at the intron 10 (int10c) site (consistent with earlier DNase 1 hypersensitivity (DHS) mapping data [34]), while Capan‐1 cells share peaks at both the intron 10 (int10a,b) and intron 11 (int11) with Caco2 cells. The function of the int10c site is not well‐characterised but it may have a structural role in the middle of the locus as it is also seen in other cell types irrespective of *CFTR* expression. Evidence of open chromatin peaks at int10a,b and int11 in Capan‐1 cells suggests the involvement of the same regulatory mechanisms and TF repertoire in pancreatic duct and intestinal epithelial cells. The int10a,b and int11 CREs have important enhancer functions in intestinal epithelial cells where they coordinately recruit FOXA1/A2, HNF1α/β, CDX2 and GATA 6 among other intestinal TFs. These TFs are required for the higher‐order chromatin looping that brings the CREs into close association with the *CFTR* promoter to drive gene expression [8, 15, 17, 28]. Also highlighted in brown on Figure 2 are open chromatin peaks in intron 20 (now intron 23), which is seen in the pancreatic lines irrespective of *CFTR* expression status (though it is very minor in Capan‐1 cells) and at +15.6 kb 3′ to the last coding base. The intron 20 site is also seen in Caco‐2 cells, where it was shown to have modest enhancer activity [34] but was not evaluated further, while the site at +15.6 kb functions as an enhancer‐blocking insulator but does not recruit CTCF [35, 36, 37]. The +15.6 kb peak is particularly evident in the BxPC‐3 line together with an additional site of open chromatin at +21.5 kb which is not seen in the other pancreatic lines. The +21.5 kb site was observed in both primary human airway (tracheal and bronchial) cells [10, 38] and in HT‐29 cells [8]. Another open chromatin peak in intron 23 (now intron 26) is seen in BxPC‐3, Capan‐1 and NP31 cells. This site was thought to be involved in the coordinate regulation of *CFTR* expression by looped enhancers in Caco‐2 cells [15, 17]. Although the intron 23 site is a very minor peak in Caco‐2 cells in Figure 2, it was shown to interact directly with the *CFTR* promoter when other intestinal enhancers in introns 1 and 11 were absent [15], suggesting enhancer capacity. Small peaks of open chromatin are also seen in Capan‐1 cells in intron 14a, 15, 17a and 18, with the 14a site also evident in BxPC‐3 and NP31 cells, the intron 15 site shared with Caco‐2 cells and the intron 17a site corresponding to an element characterised previously in Capan‐1 cells [32]. As expected, irrespective of *CFTR* expression, all the cell lines have open chromatin peaks at the TAD boundaries of the locus (blue boxes), though as observed previously, the 5′ TAD boundary is poorly accessible to the transposase used in ATAC compared to its accessibility to DNase 1 [28], while the 3′ boundary at +48.9 kb is clearly evident.

**TABLE 1:** The genomic location of intronic CREs in *CFTR* mapped to hg19 with Legacy and standard (RefSeq) nomenclature.

Chrom.	hg19 start	hg19 end	Name of DHS: Legacy	Name of CRE: RefSeq
chr7	117,119,329	117,120,116	Basal *CFTR* promoter	
chr7	117,118,149	117,120,148	2 kb *CFTR* promoter	
chr7	117,121,084	117,121,463	intron 1 CTCF	
chr7	117,129,649	117,130,749	185 + 10 kb (intron 1)	intron 1
chr7	117,145,360	117,145,679	intron 2 CTCF	
chr7	117,172,267	117,173,466	intron 4 H3K4me1	
chr7	117,212,349	117,213,649	1716 + 13.2/13.7 kb (intron/int10a,b)	intron 11 i,ii
chr7	117,222,349	117,223,049	1716 + 23 kb (intron 10c)	intron 11 iii
chr7	117,228,049	117,229,449	1811 + 0.8 kb (intron 11)	intron 12
chr7	117,240,997	117,241,300	intron 14a	intron 15
chr7	117,245,401	117,246,300	intron 15	intron 17
chr7	117,256,149	117,257,149	3600 + 1.6 kb (intron 18a)	intron 21 i
chr7	117,264,049	117,264,549	3600 + 10 kb (intron 18b)	intron 21ii
chr7	117,279,949	117,280,849	3849 + 12.5 kb (intron 19)	intron 22
chr7	117,286,112	117,286,311	intron 20	intron 23
chr7	117,305,749	117,307,149	4374 + 1.3 kb (intron 23)	intron 26

To confirm that the higher order structure of the TAD was the same in the pancreatic lines as reported previously in Caco‐2, Calu‐3 and Capan‐1 cells, among others [27, 28, 39], we performed 4C‐seq with viewpoints at the −80.1 kb 5′ TAD boundary and the *CFTR* promoter (Figure S1). All lines show strong interactions between −80.1 and +48.9 kb as expected and also peaks of interaction with the known CTCF sites at −20.9 and +6.8 kb. The promoter viewpoint showed similar interactions in the pancreatic lines and Caco‐2 cells, with marked association with the intron 23 region, consistent with the enhancer functions of that element in the pancreatic lines. However, this interaction is apparently more evident in NP31 cells than in Capan‐1 cells, despite the much higher CFTR expression in the latter. Furthermore, these 3′ interactions cannot be unequivocally attributed to the intron 23 CRE due to its proximity to the +6.8 kb CTCF site, which interacts directly with other CTCF sites across the locus [27, 37]. A 4C‐seq viewpoint designed at the intron 23 site was not informative in this regard, but a viewpoint at the intron 11 intestinal enhancer showed a peak of interactions with this site in Caco‐2 cells, consistent with cooperating enhancers [15]. Of note, the strong promoter interaction with a CRE in intron 4 in Caco‐2 cells [15] is absent from the pancreatic lines. In contrast to the strong interaction between the promoter and the −35 kb site in Calu‐3 (blue arrow in Figure S1) and other airway cells [11, 33], both NP31 and Capan‐1 cells show bimodal promoter interactions with the −20.9 kb insulator (a minor interaction in Calu‐3 cells) and a novel site 3′ to −35 kb (marked by red arrows in Figure S1).

In summary, the pancreatic lines that express *CFTR*, albeit at low levels, exhibit ATAC‐seq peaks at both the CREs that are well‐characterised in airway epithelial cells and those extensively studied in intestinal epithelial cells. The 4C‐seq data are not fully supportive of all the peaks necessarily corresponding to elements that are directly involved in driving the *CFTR* gene promoter in the pancreatic cells. Furthermore, it is not yet clear whether different cell types in these pancreatic adenocarcinoma cell lines exhibit the two open chromatin profiles or whether individual cells use both sets of CREs. Preliminary single‐cell (sc) ATAC‐seq in Capan‐1 cells would suggest the latter (data not shown).

### 3.2 *CFTR* Regulatory Elements in Primary Human Pancreatic Duct Organoids and hESC‐Derived Cholangiocyte Organoids

It is well known that adenocarcinoma cell lines such as Capan‐1, NP31 and BxPC‐3 may not faithfully recapitulate the gene expression profiles of their differentiated cells of origin and may reactivate some developmental processes. Hence, it was important to identify CREs in non‐cancerous human pancreatic duct cells, which are not readily accessible for experimentation. To achieve this, we used pancreatic duct cell organoids derived from dissected and dissociated human pancreatic ducts [19]. In parallel, we investigated organoids derived from cholangiocytes that were differentiated from human embryonic stem cells (hESC) [40]. Both cell types express CFTR (Figure S2B,C) and have many developmental similarities.

**FIGURE 3:**
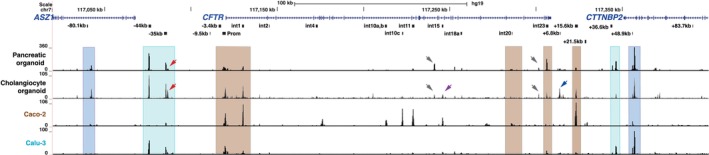
Open chromatin maps at the *CFTR* locus in primary pancreatic duct cell and cholangiocyte organoids compared to intestinal (Caco‐2) and airway (Calu‐3) epithelial cells. Open chromatin maps generated by Omni‐ATAC‐seq on pancreas organoids, HES‐derived cholangiocyte organoids and Caco‐2 and Calu‐3 cell lines are shown. Each track shows merged data from 2 technical replicates. The locations of *ASZ1, CFTR* and *CTTNBP2* are shown at the top of the figure. Key DHS identified at the *CFTR* locus in all cells, and other cell‐selective DHS of interest are marked below the gene track in black. TAD boundaries, airway‐selective DHS and intestinal DHS are marked by blue, teal and brown boxes, respectively, as described in the Figure 2 legend. Open chromatin peaks described in the results section are marked by arrowheads.

Open chromatin maps generated by ATAC for both cell types are shown in Figure 3, together with the same tracks for Caco‐2 and Calu‐3 that are utilised for comparison with the pancreatic cell lines in Figure 2. A direct comparison of the pancreatic organoid open chromatin profile and that of Capan‐1 is shown in Figure S2. Each track shows merged data from 2 biological replicates. As expected, both organoid types have open chromatin broadly across the *CFTR* promoter region, consistent with gene expression. The first clear difference between both the pancreatic and cholangiocyte organoids and the cell lines is the presence of a robust peak of open chromatin at the 5′ TAD boundary (−80.1 kb). Together with the conserved peak at the 3′ TAD boundary (+48.9 kb), we can be certain that the *CFTR* TAD we described previously [27, 28, 41] is strongly conserved in these cells. The +6.8 kb CTCF‐binding insulator (Figure 3 blue arrow) is also clear in cholangiocyte organoids. Evident in both organoid cultures are the open chromatin peaks at the intestinal CREs in introns 1 and 23, at the +15.6 kb insulator, and at uncharacterised sites in intron 14a and 21 (Figure 3, grey arrows). Minor peaks are seen in intron 11 only in the pancreatic organoids and in intron 15 only in cholangiocyte organoids (Figure 3 purple arrow), while the sites seen in Capan‐1 in introns 17a and 18 (Figures 2, Figure S2A) are almost undetectable in both organoid types. In contrast to the minor or absent open chromatin peaks at the intestinal CREs, robust ATAC‐seq peaks are evident in both pancreatic and cholangiocyte organoids at the −44 and −35 kb CREs, and at +36.6 kb only in pancreatic cells. An extra peak at ~3 kb 3 to the +36.6 kb site (+39.2 kb) is seen in both cell types and is examined in detail below. Further inspection of the −35 kb region was warranted as an additional adjacent site of open chromatin at −33 kb (Figure 3, red arrows) was seen in both organoid types but was particularly evident in cholangiocytes. We previously reported this element in 16HBE14o‐ airway epithelial cells where it coincided exactly with the central trough in a peak of H3K27ac enrichment, consistent with enhancer function [33]. It is also a minor peak of open chromatin in Calu‐3 cells in Figure 3. Thus, these organoids show open chromatin maps that are a combination of those studied previously in airway and intestinal epithelial cells, with the “airway” signature being more obvious. Though a few novel ATAC‐seq peaks are seen in the pancreatic and cholangiocyte organoids, they are very minor compared to the previously characterised sites and are of uncertain significance. These minor peaks may contain CREs that contribute to enhancer interactions and looping of the active *CFTR* locus [15] or represent alternative CREs in a rare cell type.

### 3.3 *CFTR* Regulatory Elements in Primary Human Sweat Gland Duct and Coil

**FIGURE 4:**
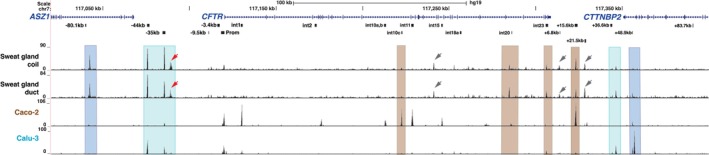
Open chromatin maps at the *CFTR* locus in human sweat gland coil and duct epithelial cells compared to intestinal (Caco‐2) and airway (Calu‐3) epithelial cells. Open chromatin maps generated by Omni‐ATAC‐seq on human sweat gland coil and duct epithelial cells and Caco‐2 and Calu‐3 cell lines are shown. Each track shows merged data from 2 technical replicates. The locations of *ASZ1, CFTR* and *CTTNBP2* are shown at the top of the figure. Key DHS identified at the *CFTR* locus in all cells, and other cell‐selective DHS of interest are marked below the gene track in black. TAD boundaries, airway selective DHS and intestinal DHS are marked by blue, teal and brown boxes, respectively, as described in the Figure 2 legend. Open chromatin peaks described in the results section are marked by grey and red arrowheads.

Another specialised ductal epithelium that has a key role in the diagnosis of CF is that of the sweat gland duct and coil. Sweat chloride levels > 60 mM/L are diagnostic of CF. To determine whether the regulatory mechanisms for the *CFTR* gene in the sweat gland were similar to those in other well‐studied epithelial cell types, we used cultures of reprogrammed, dissected human sweat gland coil and duct as described previously [21]. Though these cultures are established on irradiated mouse NIH‐3T3 cells, at confluence there are only traces of feeder cells remaining, so they do not contribute significantly to the ATAC‐seq output. The whole sweat gland cultures were shown by scRNA‐seq to include mainly ductal, secretory and myoepithelial cells [21] and to express CFTR mRNA (Figure S2D). The open chromatin profiles generated by ATAC‐seq in the duct and coil cultures separately are shown in Figure 4, where each trace shows merged data from 2 replicates. The same Caco‐2 and Calu‐3 open chromatin maps included in Figures 2 and 3 are shown below the sweat gland data for reference. As in the pancreatic and cholangiocyte organoids, a robust peak of open chromatin is seen at the 5′ TAD boundary (−80.1 kb) in both coil and duct cells; however, the peak marking the 3′ TAD boundary (+48.9 kb) is small. The promoter is open in both cell types, though the peak is more evident in the coil cells, as are the well‐characterised CREs in introns 20 and 23, +6.8 and +15.6 kb. Peaks of open chromatin are also seen in both duct‐ and coil‐derived cells in introns 14a and 21, coinciding with equivalent sites in the pancreatic and cholangiocyte organoids, and at the +21.5 kb site. The intron 14a, +6.8 and +21.5 kb sites are marked by grey arrows in Figure 4. The 21.5 kb CRE was shown previously to occur in iPSC cells differentiating towards airway epithelium [38] and to be strongly marked by H3K27Ac at the definitive endoderm (DE) stage [42]. Possibly this site is associated with the reprogramming protocols that are used in both culture systems. Both the +36.6 kb site and +39.2 kb sites (Figure 4, teal box) are open in the sweat gland duct and coil cultures, with the 3′ site being more evident. A novel and broad (bimodal) ATAC‐seq peak is seen in both cell types (marked by a red arrow in Figure 4) 3′ to both the −35 and −33 kb CREs at ~−31 kb.

### 3.4 Histone Modification at CREs in NP31 and Capan‐1 Cells

**FIGURE 5:**
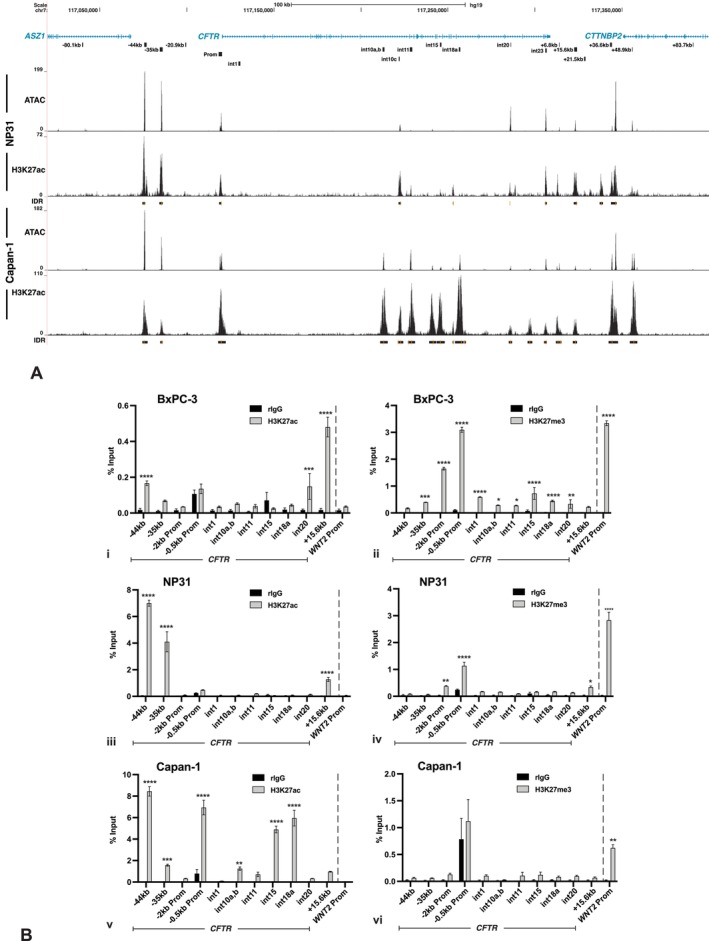
*CFTR* expression levels correlate with active and repressive histone modifications at the gene promoter and at specific CREs across the locus in pancreatic adenocarcinoma cell lines. (A) Map of active histone (H3K27Ac) modifications generated by ChIP‐seq, aligned to the same ATAC‐seq peak tracks shown in Figure 2 in NP31 and Capan‐1 cells. An individual ChIP‐seq experiment is illustrated for each line, below which are shown IDR peaks from replicate experiments. (B) ChIP for H3K27ac in (i) BxPC‐3; (iii) NP31; (v) Capan‐1 cells. ChIP for H3K27me3 in (ii) BxPC‐3; (iv) NP31; (vi) Capan‐1. All ChIP datasets are normalised to percent input. *n* = 3. Enrichment level statistics: ****p < 0.0001; ***p < 0.001; **p < 0.01; *p < 0.05 using a two‐way ANOVA with Sidak's multiple comparison test.

Next, to determine which of the CREs were likely enhancers in the Capan‐1 and NP31 cells, we performed ChIP‐seq for the active histone mark H3K27ac (Figure 5A). Both cell lines had peaks of H3K27ac at the *CFTR* promoter, indicative of an active promoter, although the enrichment in NP31 was much lower than in Capan‐1, consistent with the different *CFTR* expression levels in the two lines. Substantial H3K27Ac was also seen in both cell lines at the −44 and −35 kb CREs upstream of the promoter [10, 13, 14], at previously defined CREs in introns 10 (int10c), 20 and 23 (Figure 5A, S3A, S3A) [17, 34, 43] and downstream of the gene at +6.8, +15.6, + 36.6 and +48.9 kb. A second peak 3′ to the +36.6 kb site at +~39.2 kb (hg19:117,346,450) was also noted in both cell lines, and the characteristic distribution of H3K27ac flanking each of these sites is seen in Figure S3B. Of note, the map location of the +36.6 kb site, which was originally identified by DNAse‐seq in primary human tracheal epithelial (HTE), mixed tracheal and bronchial (NHBE) epithelial cells and Calu‐3 cells [10], appears slightly 5′ to the CRE in the pancreatic cell lines, where the apex of the ATAC‐seq open chromatin peak is at ~+37.2 kb (hg19:117,344,349), though it is likely the same element. Several peaks of H3K27ac enrichment are only seen in Capan‐1 cells and correspond to several intestinal CREs, including those in intron 1, intron 10 (int10a, b), intron 15 (int 15), intron 18 (int18a) and an additional site in intron 21 that is not well‐characterised.

To validate the ChIP‐seq data and extend the analysis of histone marks to the CFTR‐negative BxPC‐3 pancreatic line, we performed ChIP‐qPCR for H3K27Ac and H3K27me3 using primer sets at −44, −35, −2 and −0.5 kb promoter regions, at CREs in introns 1, 10 (int10a,b), 11, 15, 18 (int18a) and 20, and at +15.6 kb (Figure 5B). The *WNT2* promoter was used as a control for negative histone modification since it shows a strong ATAC‐seq peak in the pancreatic cell lines where the gene is not expressed. Significant H3K27ac enrichment was evident at the −44 and −35 kb CREs in both NP31 and Capan‐1 cells, though only at −44 kb in BxPC‐3 cells (Figure 5Bi,iii,v). The relative levels of enrichment in the pancreatic lines were consistent with the ATAC‐seq peaks, since these were evident in all three lines (Figure 2), but not with the level of *CFTR* expression when comparing NP31 and Capan‐1, as the latter expresses substantially more CFTR protein. Next, considering H3K27ac levels at known intestinal intronic enhancers and ATAC‐seq peaks across the locus: though no statistically significant enrichment is seen in NP31 and BxPC‐3, the Capan‐1 line shows low but significant levels of the active histone mark at CREs in intron 10 (int10a,b) and much higher enrichment at the intron 15 and intron 18 (int18a) sites, suggesting that these sites may serve as enhancers of *CFTR* expression in Capan‐1 cells. Significant enrichment of H3K27ac was also seen at the +15.6 kb insulator only in BxPC‐3 and NP31 cells. A closer correlation was seen between occupancy of the active histone mark at the *CFTR* gene basal promoter (−0.5 kb) and *CFTR* expression in the pancreatic lines, where only Capan‐1 cells showed substantial enrichment. Next, we examined occupancy of the negative histone mark (Figure 5Bii,iv,vi) and observed no enrichment of H3K27me3 at the −44 kb site in any of the cell lines. However, there was minor, though statistically significant, occupancy of H3K27me3 at the basal promoter and broader promoter regions (−2 kb CFTR promoter) in NP31 cells consistent with low *CFTR* expression and substantial occupancy at both promoter regions in BxPC‐3 reflecting the lack of *CFTR* expression in these cells. Of note, many of the CREs, including the −35 kb and intron 1 enhancers, together with open chromatin peaks in introns 1, 10 (int10a,b), 11, 15, 18 (int18a) and 20, showed statistically significant enrichment of H3K27me3 in BxPC‐3 cells (Figure 5Bii). The minor enrichment of H3K27me3 at the basal promoter in Capan‐1 cells is not significantly above rIgG alone. In summary, the profile of histone modifications across the *CFTR* locus is strongly indicative of histone methylation at the gene promoter playing a major role in silencing *CFTR* expression in several pancreatic cell lines, with only Capan‐1 cells, which do express *CFTR*, showing substantial histone acetylation at this site. The relative levels of H3K27ac enrichment at CREs across the locus in Capan‐1 and NP31 cells (only detected by ChIP‐seq) also suggest that multiple intronic sites may cooperate in maintaining gene expression as is seen in other cell types [8, 15, 28] and, moreover, that CREs at the common intestinal and unique pancreatic peaks of open chromatin both play a key role.

### 3.5 The Transcription Factors Predicted to Drive *CFTR* Expression in the Pancreatic Cell Lines, Cholangiocyte Organoids and Sweat Gland Epithelial Cells

In earlier work we predicted transcription factors (TFs) that might bind to pancreatic cell CREs, based upon DNase‐1 footprinting and electrophoretic mobility shift assays, and identified PBX homeobox 1 (PBX1) as an interacting factor by ChIP‐qPCR [32]. To extend these predictions, we first used Transfac 2.0 (genexplain.com) to search for predicted transcription factor binding sites in the core of the CREs in introns 15, 18 and 20, seen in Capan‐1 cells. The intron 15 element contains multiple predicted binding sites for CDX2, Krüppel‐like factors 4 and 6 (KLF4 and KLF6), a glucocorticoid response element (GRE), and a single CAAT enhancer‐binding protein site which is very close to the 3′ end of the peak and so unlikely to be of biological significance (Figure S4). Consistent with the presence of the intron 15 site in Caco‐2 cells, CDX2 is known to be a major component of the transcriptional network in intestinal epithelial cells and to directly regulate *CFTR* [16, 17]. The KLF family of transcription factors, including both KLF4 and KLF6, have a well‐established role in cancer progression (reviewed in [44]), and we showed direct regulation of *CFTR* by KLF5 in airway epithelial cells where this factor has a critical role in wound repair and innate immunity [11, 45]. The intron 18 element contains multiple motifs for FOX family factor binding [46], including specifically FOXC, as well as for HNF‐like factors, CDX2, C/EBP, a paired estrogen‐response (ER) element site, and one central site for ISL LIM homeobox 1 (ISL1), a factor known to drive insulin gene expression and to be important for lineage determination in the pancreas [47] (Figure S5). Broadly, the same factors are predicted *in silico* to bind within the intron 20 CRE (Figure S6) in addition to activating transcription factor 2 (ATF‐2) group factors and SOX‐related factors.

Next, addressing predicted TF binding sites in the primary pancreatic organoids and hESC‐derived cholangiocyte organoids open chromatin peaks, we looked specifically at the −33 kb site, which has two sub‐peaks in pancreatic organoids and is always seen in conjunction with the well‐characterised site at −35 kb [33]. The −33 kb sub‐peaks both have predicted binding sites for AP‐2 [48] and inhibitor of growth family member 4 (ING4) [49] (Figure S7).

Lastly, we inspected the novel peak of open chromatin seen at −31 kb only in the sweat gland duct and coil for predicted TF binding motifs. Analysis of the 120 bp DNA sequence in the central trough of this peak (Figure S8 (AliBaba2.1)) showed predicted binding sites for multiple TFs, including the estrogen receptor (ER), nuclear factor kappa B (NFKB), retinoic acid receptor alpha (RXR‐alpha), HNF1 and CREB binding protein (CREBPB), all TFs that have been implicated at other CFTR CREs. Analysis of the 120 bp sequence on a second TF binding motif prediction platform (TRAP [50]) confirmed these predictions, with the exception of HNF4 instead of HNF1 (data not shown). This site warrants further investigation in the context of the sweat gland epithelial transcriptome.

Subsequently, instead of investigating predicted TF binding at individual open chromatin peaks in the *CFTR* locus, we evaluated overrepresented TF binding sites predicted in open chromatin peaks genome‐wide in pancreatic and cholangiocyte organoids and sweat gland duct and coil cultures using the HOMER 2 v5.1 pipeline [26]. First, considering pancreatic (Table 2) and cholangiocyte (Table 3) organoids and examining IDR peaks shared between the two biological replicates of each cell type, we see substantial similarity in the predictions for both. Predicted overrepresented TF binding sites include EHF, HNF1β, HNF4α (cholangiocyte only), NRF1, the FOX:Ebox (Forkhead, bHLH) and NFY, in addition to very common factors that appear on many HOMER analyses (e.g., AP‐1 and Sp1/2) (Table 2). Of note, all these TFs were identified in the transcriptional networks of other epithelial cells previously, and several were predicted or shown to have a role in *CFTR* expression in other epithelial cells (reviewed in [51]) [13, 14, 27, 30, 43, 52, 53, 54]. The top 10 most overrepresented de novo motifs, when analysing the IDR peaks shared between the two biological replicates of sweat gland coil and sweat gland duct, are rather similar (Tables 4 and 5). In addition to common TF motifs (such as Fra1 and AP‐1), Sp2, RUNX1, TEAD3, NFKβ‐p65 and NRF1 motifs are overrepresented in cells from both regions. Of note, RUNX1 has a well‐established role in the skin, where it plays a critical role in hair follicles [55], and it may have an important role in androgen regulation of gene expression in the human epididymis epithelium [56], another site of *CFTR* expression. CCAAT/enhancer‐binding protein (CEBP) family members [57] CEBPε and CEBPẟ motifs are overrepresented in open chromatin peaks in coil and duct, respectively. Furthermore, another CEBP component, CEBPβ, is involved in CFTR regulation both via the promoter and the −35 kb enhancer [33, 58].

**TABLE 2:** HOMER analysis of open chromatin peaks in pancreatic organoids. The top 10 most overrepresented *de novo* motifs, when analysing the IDR peaks shared between the two biological replicates, are shown.

Rank	log_*p*_value	% targets	% background	best match/details	best_match
1	−9180	32.23	10.1	Atf3(bZIP)/GBM‐ATF3‐ChIP‐Seq(GSE33912)/Homer(0.988)	Atf3(bZIP)
2	−2221	36.38	23.18	EHF(ETS)/LoVo‐EHF‐ChIP‐Seq(GSE49402)/Homer(0.971)	EHF(ETS)
3	−1399	23.83	14.86	Sp2(Zf)/HEK293‐Sp2.eGFP‐ChIP‐Seq(Encode)/Homer(0.952)	Sp2(Zf)
4	−1111	15.21	8.74	Fox:Ebox(Forkhead,bHLH)/Panc1‐Foxa2‐ChIP‐Seq(GSE47459)/Homer(0.952)	Fox:Ebox(Forkhead,bHLH)
5	−916.8	6.64	2.92	BORIS(Zf)/K562‐CTCFL‐ChIP‐Seq(GSE32465)/Homer(0.925)	BORIS(Zf)
6	−879.4	10.6	5.77	HNF1b(Homeobox)/PDAC‐HNF1B‐ChIP‐Seq(GSE64557)/Homer(0.847)	HNF1b(Homeobox)
7	−728	19.75	13.61	POL013.1_MED‐1/Jaspar(0.764)	POL013.1_MED‐1
8	−710.5	20.01	13.89	NRF1/MA0506.1/Jaspar(0.954)	NRF1
9	−566.8	21.09	15.44	E2F(E2F)/Hela‐CellCycle‐Expression/Homer(0.901)	E2F(E2F)
10	−548.1	20.86	15.32	STP3/MA0396.1/Jaspar(0.730)	STP3

**TABLE 3:** HOMER analysis of open chromatin peaks in cholangiocyte organoids. The top 10 most overrepresented *de novo* motifs, when analysing the IDR peaks shared between the two biological replicates, are shown.

Rank	log_*p*_value	% targets	% background	best_match/details	best_match
1	−17,580	30.62	8.26	AP‐1(bZIP)/ThioMac‐PU.1‐ChIP‐Seq(GSE21512)/Homer(0.993)	AP‐1(bZIP)
2	−4374	43.17	28.12	EHF(ETS)/LoVo‐EHF‐ChIP‐Seq(GSE49402)/Homer(0.975)	EHF(ETS)
3	−3917	19.79	9.7	Sp1(Zf)/Promoter/Homer(0.967)	Sp1(Zf)
4	−3271	7.37	2.21	HNF1b(Homeobox)/PDAC‐HNF1B‐ChIP‐Seq(GSE64557)/Homer(0.986)	HNF1b(Homeobox)
5	−3264	8.21	2.67	HNF4a(NR),DR1/HepG2‐HNF4a‐ChIP‐Seq(GSE25021)/Homer(0.953)	HNF4a(NR),DR1
6	−2723	10.13	4.18	BORIS(Zf)/K562‐CTCFL‐ChIP‐Seq(GSE32465)/Homer(0.926)	BORIS(Zf)
7	−2129	7.87	3.21	NRF1(NRF)/MCF7‐NRF1‐ChIP‐Seq(Unpublished)/Homer(0.990)	NRF1(NRF)
8	−2014	13.69	7.38	Fox:Ebox(Forkhead,bHLH)/Panc1‐Foxa2‐ChIP‐Seq(GSE47459)/Homer(0.925)	Fox:Ebox(Forkhead,bHLH)
9	−1768	10.62	5.43	Atf1(bZIP)/K562‐ATF1‐ChIP‐Seq(GSE31477)/Homer(0.966)	Atf1(bZIP)
10	−1762	8.58	4.01	NFY(CCAAT)/Promoter/Homer(0.946)	NFY(CCAAT)

**TABLE 4:** HOMER analysis of open chromatin peaks in sweat gland coil cells. The top 10 most overrepresented *de novo* motifs, when analysing the IDR peaks shared between the two biological replicates, are shown.

Rank	log_*p*_value	% targets	% background	best_match/details	best_match
1	−33,350	39.96	9.05	Fra1(bZIP)/BT549‐Fra1‐ChIP‐Seq(GSE46166)/Homer(0.995)	Fra1(bZIP)
2	−4728	25.62	13.81	ETV4(ETS)/HepG2‐ETV4‐ChIP‐Seq(ENCODE)/Homer(0.962)	ETV4(ETS)
3	−4209	19.45	9.69	Sp2(Zf)/HEK293‐Sp2.eGFP‐ChIP‐Seq(Encode)/Homer(0.971)	Sp2(Zf)
4	−3231	14.86	7.3	RUNX1(Runt)/Jurkat‐RUNX1‐ChIP‐Seq(GSE29180)/Homer(0.973)	RUNX1(Runt)
5	−2890	18	9.97	TEAD3/MA0808.1/Jaspar(0.967)	TEAD3
6	−2598	18.5	10.71	CEBPE/MA0837.1/Jaspar(0.925)	CEBPE
7	−2042	20.4	13.02	NFkB‐p65‐Rel(RHD)/ThioMac‐LPS‐Expression(GSE23622)/Homer(0.947)	NFkB‐p65‐Rel(RHD)
8	−1842	5.26	1.99	NRF1(NRF)/MCF7‐NRF1‐ChIP‐Seq(Unpublished)/Homer(0.985)	NRF1(NRF)
9	−1519	28.15	20.71	TFAP2C/MA0524.2/Jaspar(0.900)	TFAP2C
10	−1512	18.32	12.19	STB2(MacIsaac)/Yeast(0.748)	STB2(MacIsaac)

**TABLE 5:** HOMER analysis of open chromatin peaks in sweat gland duct cells. The top 10 most overrepresented *de novo* motifs, when analysing the IDR peaks shared between the two biological replicates, are shown.

Rank	log_*p*_value	% targets	% background	best_match/details	best_match
1	−38,200	43.45	9.46	AP‐1(bZIP)/ThioMac‐PU.1‐ChIP‐Seq(GSE21512)/Homer(0.990)	AP‐1(bZIP)
2	−5298	32.77	18.83	ETS1(ETS)/Jurkat‐ETS1‐ChIP‐Seq(GSE17954)/Homer(0.980)	ETS1(ETS)
3	−3886	15.85	7.4	Sp2(Zf)/HEK293‐Sp2.eGFP‐ChIP‐Seq(Encode)/Homer(0.953)	Sp2(Zf)
4	−2384	8.26	3.49	CEBPD/MA0836.2/Jaspar(0.912)	CEBPD
5	−2285	12.91	6.81	Atf2(bZIP)/3T3L1‐Atf2‐ChIP‐Seq(GSE56872)/Homer(0.957)	Atf2(bZIP)
6	−2144	9.4	4.44	NFkB‐p65(RHD)/GM12787‐p65‐ChIP‐Seq(GSE19485)/Homer(0.950)	NFkB‐p65(RHD)
7	−1993	8.34	3.85	BORIS(Zf)/K562‐CTCFL‐ChIP‐Seq(GSE32465)/Homer(0.891)	BORIS(Zf)
8	−1905	14.84	8.74	RUNX(Runt)/HPC7‐Runx1‐ChIP‐Seq(GSE22178)/Homer(0.966)	RUNX(Runt)
9	−1713	5.17	2.01	NRF1(NRF)/MCF7‐NRF1‐ChIP‐Seq(Unpublished)/Homer(0.998)	NRF1(NRF)
10	−1679	20.69	13.86	TEAD3/MA0808.1/Jaspar(0.946)	TEAD3

## 4 Discussion

For many years, the *CFTR* gene was a paradigm for deciphering regulatory mechanisms in large genes with key control elements lying outside the gene promoter. Its prominence coincided with the need to define the elements responsible for tissue‐specific and temporal regulation of this disease‐associated gene to guide the development of new therapeutics. Progress was enabled by the development of new functional genomics technologies and by advances in primary cell and organoid culture systems, which generated novel reagents for studying *CFTR*. Shortly after *CFTR* was cloned [59, 60, 61], multiple studies suggested that the *CFTR* promoter region lacked tissue‐specific control elements [62, 63]. In early work, before the human genome was fully sequenced, the use of DHS mapping provided the first evidence that *CFTR* regulatory elements might lie within introns [64]. Subsequent efforts of the ENCODE consortium [65] and studies on many other genes enabled not only the identification and characterisation of CREs but also the integration of higher‐order chromatin structure, transcription factor recruitment, and histone modification into the *CFTR* locus regulatory mechanisms (reviewed in [7]).

Multiple investigators have now shown and validated the *CFTR* TAD, the existence of an extensive set of CREs flanking the locus and within introns, and recruitment of those CREs to the gene promoter in a temporal and spatial manner [8, 9, 15, 28, 33, 39, 66]. Initial studies showed that intronic CREs recruiting intestinal transcription factors such as HNF1α, FOXA2 and CDX2 were the dominant elements in driving *CFTR* expression in the intestinal epithelium. In contrast, CREs upstream of the promoter and downstream of the gene were found to be critical in regulating *CFTR* expression in the airway epithelium [10, 33]. However, an exception to these clear distinctions was provided by DNase‐seq analysis of primary human epididymis epithelial cells [8], which clearly showed DHS corresponding to both intestinal and airway CREs, validated more recently by ATAC‐seq [18]. In light of these observations in the epididymis, we were not surprised to find that other cell types also have open chromatin at both the intestinal and airway sets of CREs. Here we describe profiles of pancreatic duct, bile duct and sweat gland duct and coil epithelium, which have many chromatin features at the *CFTR* locus in common with the epididymis epithelium but also some unique cell‐selective elements. However, a question now arises over whether the detection of a peak of open chromatin at a known CRE always correlates with an activating role in *CFTR* expression. This applies particularly to the data presented here on open chromatin peaks in the pancreatic cell lines BxPC‐3, NP31 and Capan‐1, which express no, very low, or low levels of CFTR, respectively. All three lines have robust open chromatin peaks at the −44 and −35 kb CREs that are known to be enhancers in airway epithelial cells, and both sites are marked by peaks of histone acetylation in ChIP‐seq data from NP31 and Capan‐1 cells. These results are confirmed by ChIP‐qPCR, although the relative enrichment of H3K27ac at the −35 kb site does not correlate with CFTR expression levels in these two lines, since it is higher in NP31 cells. Of note, there is no significant enrichment of H3K27ac at the −35 kb sites in BxPC‐3 cells. Considering CREs that are known to be enhancers in intestinal epithelial cells, including those in intron 11 and intron 20, intron 11 exhibits a small peak of open chromatin only in Capan‐1 cells, while the intron 20 element is almost undetectable in Capan‐1 cells and open in both NP31 and BxPC‐3 cells. Neither of these sites shows significant enrichment of H3K27ac in Capan‐1 or NP31 cells by ChIP‐qPCR, although the intron 20 site is enriched in BxPC‐3 cells. These observations suggest that though these sites are accessible to the transposase used in ATAC‐seq and hence can be described as open chromatin, they may be in a poised rather than an active state in the pancreatic lines. Consistent with this interpretation are the 4C‐seq data, which do not show clear interactions between the CFTR promoter and the CREs in introns 11 and 20. Conversely, the 4C‐seq data suggest the −44 and −35 kb regions are in close association with the *CFTR* promoter, although the profile of interactions is different from that seen in Calu‐3 cells, airway cells that express abundant *CFTR*. Both pancreatic lines exhibit a strong peak of interaction between the *CFTR* promoter and the −20.9 kb insulator in addition to the −35 kb region, while the −20.9 kb interaction is minor in Calu‐3 cells. Recruitment of this enhancer‐blocking insulator might contribute to the low levels of *CFTR* expression in the pancreatic lines.

An important observation on the mechanisms of low or absent *CFTR* expression in the pancreatic cell lines is the presence of the negative histone mark H3K27me3. In earlier work we examined the DNA methylation status of the *CFTR* promoter in multiple cell lines and showed that it is rarely fully methylated and is generally unmethylated, irrespective of promoter activity, suggesting this was not a key regulatory mechanism [67]. However, the data we present here clearly demonstrate histone methylation at the basal and extended (−2 kb) *CFTR* promoter in BxPC‐3 cells, consistent with promoter silencing in this line, (and, to a lesser extent, in NP31 cells correlated with very low CFTR). This result suggests that the accessibility of CREs alone may not be a reliable guide to their utilisation in specific cell types and that multiple layers of regulatory mechanisms, perhaps including the availability of specific TF complexes, guide the activation or repression of *CFTR* expression. Another relevant mechanistic component is the co‐operation between multiple regulatory elements within the same cell which is documented in several studies [8, 10, 28]. Moreover, we showed earlier that the removal of individual CREs from the locus resulted in the activation of other potential elements [15], suggesting enhancer redundancy and cross‐talk. For example, the removal of the intron 1 and/or intron 11 CRE in Caco‐2 cells promoted inclusion of the intron 23 (intron 26) site in the looping interactions with the *CFTR* gene promoter [15].

Lastly, we focus on the apparently novel peaks of open chromatin seen in the pancreatic and cholangiocyte organoids in introns 14a and 21 and in the sweat gland duct and coil at −31 kb. It seems likely that if these peaks mark cell‐specific CREs, they are components of a coordinated enhancer network driving *CFTR* expression in these cells. Further characterisation of these sites could reveal the precise TFs that occupy these elements, which may show some cell‐type selectivity and hence be of interest in documenting the transcriptional networks in these rare cell types. However, these studies may be unlikely to reveal any new critical CREs for *CFTR* but rather define “shadow enhancers” (reviewed in [68]) or accessory enhancers held in reserve until a biological need arises. One exception might be hormone‐dependent CREs that are active only in unique cell types, for example, the AR‐dependent CRE at −9.5 kb that is seen predominantly in primary human epididymis epithelial cells [18].

**FIGURE 6:**
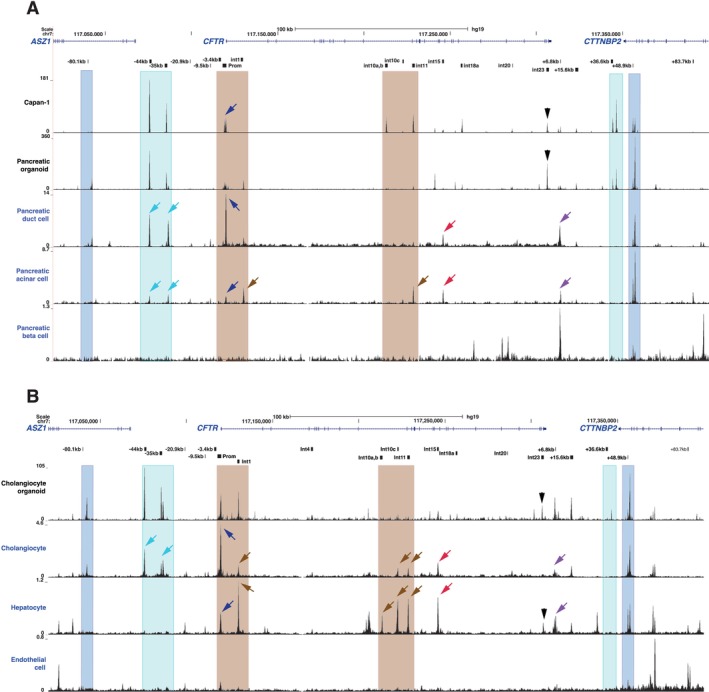
Open chromatin maps at the *CFTR* locus in key cell types from human pancreas and liver tissue by snATAC‐seq. (A) Pancreatic duct cell, acinar cell and beta cell snATAC‐seq compared to ATAC‐seq from Capan‐1 cells and pancreatic organoids (Figure 2). (B) Cholangiocyte, hepatocyte and liver endothelial cell snATAC‐seq compared to ATAC‐seq from cholangiocyte organoids (Figure 3). Data from [70, 71]. Key: Blue box: TAD boundary; teal box and arrows: Airway CREs; brown box and arrows: intestinal CREs, as described in the discussion. Blue arrows: Promoter; red arrows: Intron 15 CRE; purple arrows: +6.8 kb CTCF‐binding insulator element. Legacy nomenclature.

The particular need to document CREs controlling the *CFTR* gene to inform novel gene‐based therapeutics would seem to be largely satisfied by the current and existing data in different epithelial cell types, though unanswered questions remain:

First, it is not clear whether the combination of CREs observed in human pancreatic organoids (and in human primary epididymis cells) reflects the open chromatin profile at the *CFTR* locus in all *CFTR*‐expressing cells or whether different CRE subsets are associated with more than one cell type within the epithelium. Ultimately, scRNA‐seq in combination with single nuclear (sn) ATAC‐seq should resolve this question. These protocols are already powerful in distinguishing multiple cell types in the pancreas [69], though the current level of resolution is not yet sufficient to separate very similar cell types. We examined this question further by comparing snATAC‐seq data from ductal and acinar cells in whole pancreas tissue [70] and from cholangiocytes and hepatocytes in whole liver tissue [71] (Figure 6A,B). In pancreatic duct cells that express abundant *CFTR*, chromatin at the −44 and −35 kb sites (Figure 6A, teal arrows) is highly accessible, as is the promoter, while elements in introns 1, 10 and 11 are not. In contrast, in acinar cells which express little or no *CFTR* in the scRNA‐seq data, open chromatin peaks are evident at these intronic elements, while both the −44 and 35 kb sites and the promoter are poorly accessible compared to duct cells. The intron 15 element (Figure 6A, red arrows) is an open chromatin peak in both cell types, as is the CTCF‐binding insulator element at +6.8 kb [37]. Also of note is the intron 23 CRE (black arrow in Figure 6A), which, although a feature of both Capan‐1 cells and the pancreatic organoids, is not seen in either pancreatic duct or acinar cells. Pancreatic beta cells provide a negative control for the inactive *CFTR* locus. Although these data provide robust evidence for the recruitment of different sets of CFTR regulatory elements in the pancreatic duct and acinar cell compartments, further analysis is needed to distinguish the multiple types of duct cells that likely express variant levels of *CFTR*. Next, examining snATAC‐seq data in liver (Figure 6B), in cholangiocytes high *CFTR* expression is associated with substantial peaks of open chromatin at −44 kb, −35 kb, and at the promoter, and lesser peaks at the intronic elements (int1, int10c and int11), suggesting that all these elements may be involved in gene expression. In contrast, hepatocytes, which have no *CFTR* activity, have no snATAC‐seq peaks at −44 kb or −35 kb, while strong peaks are seen at the intronic sites (int1, int10a,b, int10c, int11) though the promoter peak is small. As in the pancreas, the int15 site is open in both cell types, though the int23 site is only open in hepatocytes. Hence, in cholangiocytes, activation of the CREs at −44 and −35 kb may be critical for high *CFTR* levels, a hypothesis that can be tested by future analysis including linking open chromatin sites to gene promoters, for example with activity‐by‐contact (ABC) analysis [72].

A second important unanswered question relates to the mechanisms of *CFTR* regulation in the rare, high CFTR cells in the airway (pulmonary ionocytes) and in the epididymis (clear cells), which share a very similar transcriptome [73, 74]. Direct observations on active regulation of the *CFTR* gene in these and other differentiated cell types will likely be delivered by future advances in single‐cell functional genomics.

## Author Contributions

**Ayushi Umrigar:** data curation (equal), formal analysis (equal), investigation (equal), methodology (equal), validation (equal). **Katreya Lovrenert:** data curation (equal), formal analysis (equal), investigation (supporting), methodology (equal), resources (equal), software (lead), validation (equal). **Jenny L. Kerschner:** data curation (supporting), formal analysis (supporting), investigation (supporting), methodology (supporting), validation (supporting), writing – review and editing (supporting). **Neeraj Sharma:** funding acquisition (supporting), resources (equal), writing – review and editing (supporting). **Britney Tian:** resources (equal). **Rebecca Melton:** formal analysis (equal). **Kyu Shik Mun:** resources (equal). **Nirbhayaditya Vaghela:** investigation (supporting). **Mina Ogawa:** resources (equal). **Gedge D. Rosson:** resources (supporting). **Shinichiro Ogawa:** resources (equal). **Anjaparavanda P. Naren:** funding acquisition (supporting), resources (equal). **Kyle J. Gaulton:** formal analysis (equal). **Shih‐Hsing Leir:** investigation (supporting), methodology (supporting), writing – review and editing (supporting). **Ann Harris:** conceptualization (lead), formal analysis (lead), funding acquisition (lead), investigation (equal), methodology (equal), project administration (lead), supervision (lead), validation (equal), visualization (lead), writing – original draft (lead), writing – review and editing (lead).

## Conflicts of Interest

K.J.G. has consulted for Genentech and received honoraria from Pfizer, holds stock in Neurocrine Biosciences, and his spouse is employed by Altos Labs Inc.

## Supporting information


**Figure S1–S8:** jcmm70751‐sup‐0001‐FigureS1‐S8.pdf.

**Table S1–S2:** jcmm70751‐sup‐0002‐TableS1‐S2.docx.

**Data S1:** jcmm70751‐sup‐0003‐DataS1.docx.

## Data Availability

Genome‐wide data are deposited at GEO: GSE283992.
