# Supplementary material for: CFTR Gene Regulation in Human Pancreatic Duct, Bile Duct and Sweat Gland Epithelial Cells
Source: J Cell Mol Med. 2025 Aug 10;29(15):e70751. doi: 10.1111/jcmm.70751 (PMC12336291; doi:10.1111/jcmm.70751)
Supplement: Supplementary file 1 — Figure S1–S8: jcmm70751‐sup‐0001‐FigureS1‐S8.pdf. [file JCMM-29-e70751-s002.pdf]

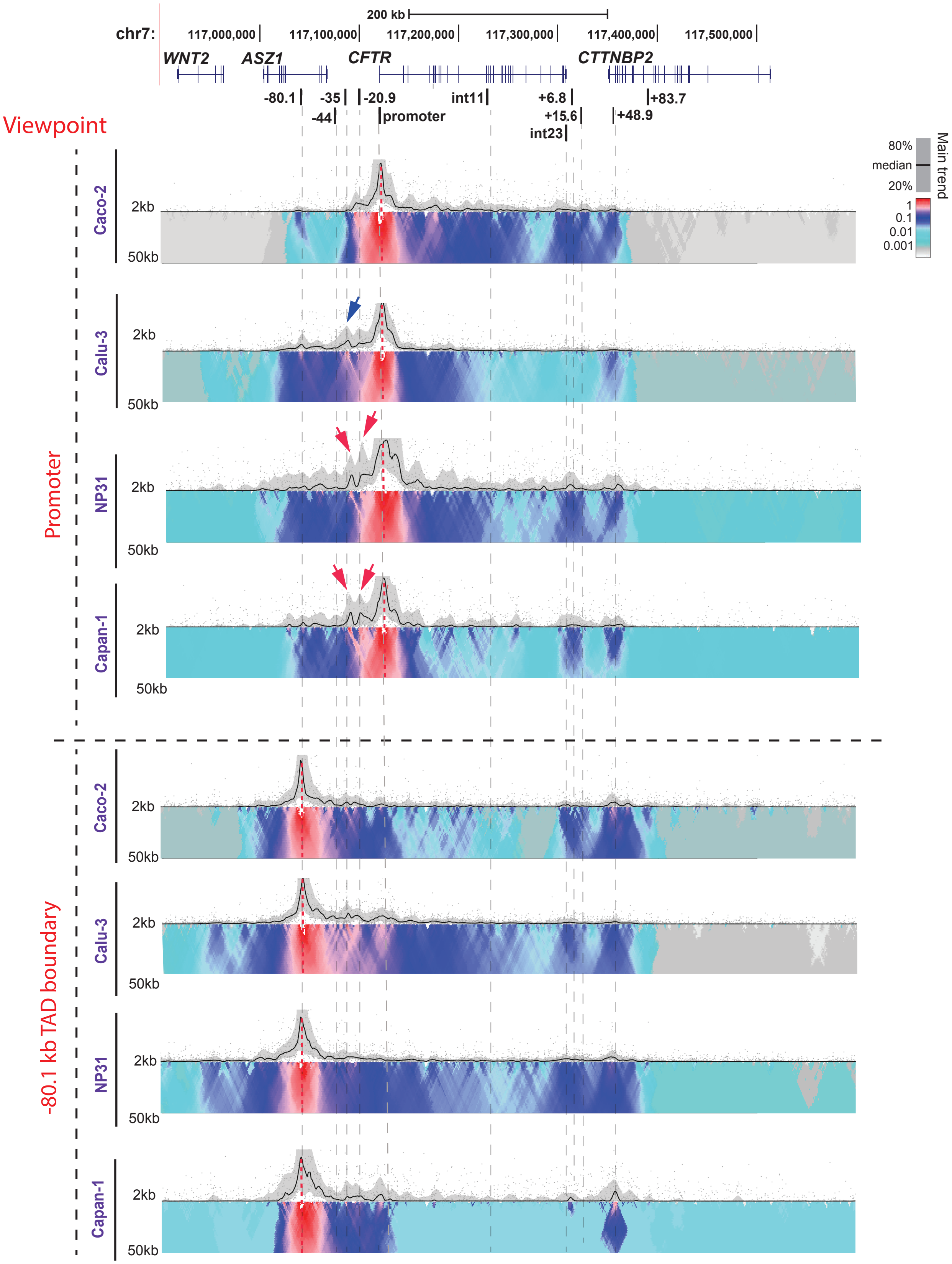

Figure S1: Pancreatic adenocarcinoma cell chromatin architecture at the *CFTR* locus: 4C-seq data are shown for Caco-2, Calu-3, Capan-1 and NP31 cell lines. At the top the genomic location of *CFTR* and adjacent genes on chromosome 7 are shown together with known *CFTR cis*-regulatory elements. Below, 4C-seq data are shown for each cell line using viewpoints at the *CFTR* promoter (upper) and the -80.1kb 5' TAD boundary (lower). The 4C-seq data show the main trend of contact profile using a 5-kb window size as a black line above the domainogram. Relative interactions are normalized to the strongest point (which is set to 1) within each panel. The domainogram uses color-coded intensity values to show relative interactions with window sizes varying from 2 to 50 kb. Here, red denotes the strongest interactions and dark blue, through turquoise, to gray represent decreasing frequencies.

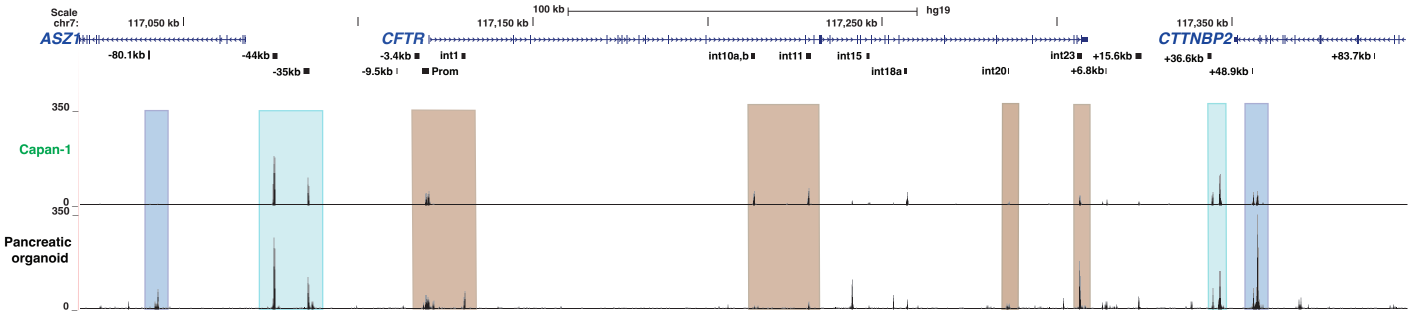

Figure S2A

Open chromatin maps at the *CFTR* locus in the Capan-1 pancreatic adenocarcinoma cell line compared to pancreatic duct cell organoids. Open chromatin maps generated by Omni-ATAC-seq are shown. Each track shows merged data from 2 technical replicates. The location of *ASZ1*, *CFTR* and *CTTNBP2* are shown at the top of the figure. Key DHS identified at the *CFTR* locus in all cells, and other cell-selective DHS of interest are marked below the gene track in black. This manuscript uses legacy nomenclature for the CFTR gene to be consistent with our earlier work (see Table 1 for conversion to Refseq). TAD boundaries are shown by blue boxes at -80.1 kb and +48.9kb. Airway selective DHS at -35 and -44 kb and +36.6 kb are shown by teal boxes. Intestinal selective DHS at intron 10a,b, intron 11, intron 20, intron 23, and +15.6 kb are shown by brown boxes.

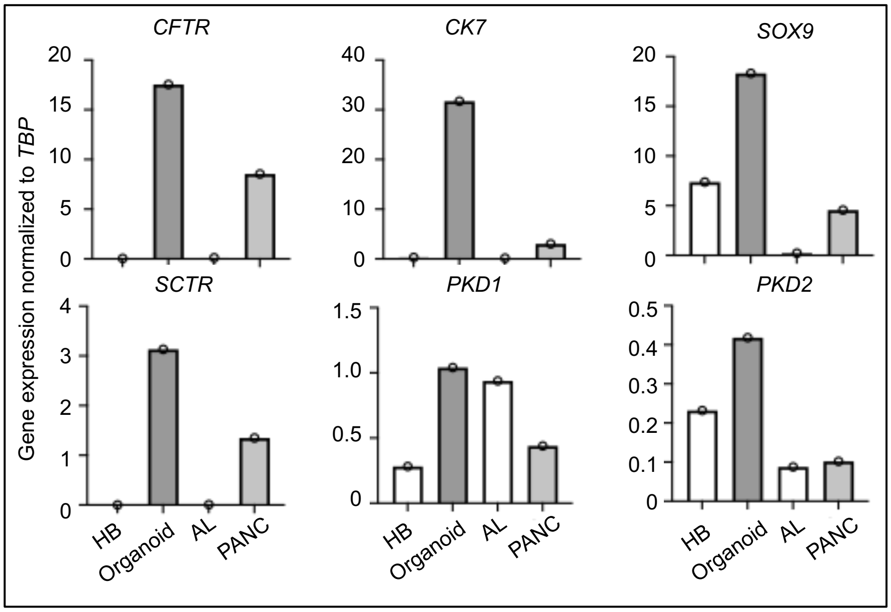

Figure S2B. Functional maturation markers in human pluripotent stem cell-derived cholangiocyte organoids. RT-qPCR of RNA from HB, hepatoblasts (hepatic progenitor cells, the progenitor cell before cholangiocyte differentiation); cholangiocyte organoids; AL, adult human liver; PANC, adult human pancreas. Gene expression is normalized to TATA-box binding protein (*TBP*). Key: *CFTR* - cystic fibrosis transmembrane conductance regulator; *CK7* - cytokeratin 7; *SOX9* - SRY-box transcription factor 9; *SCTR* - secretin transmembrane receptor; *PKD1* - polycystin 1; *PKD2* - polycystin 2. Protocols and primers are shown in the Supplementary methods.

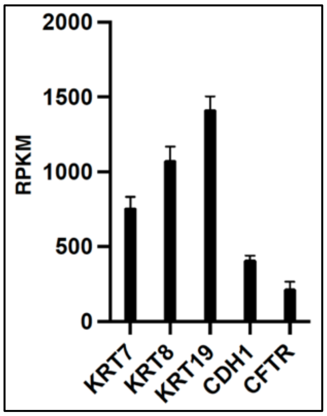

Figure S2C. Pancreatic duct cell marker gene expression in pancreatic organoids. Data from bulk RNA-seq of 4 replicates of pancreatic organoid cultures (RPKM values as described in ref 17, PMID 31311920). Key: keratin 7-KRT7; keratin 8 - KRT8; keratin 19 - KRT19; CDH1- cadherin 1; CFTR as in Fig. 2B.

Figure S2D. *CFTR* expression in eccrine sweat gland (ESG) cultures compared to the Calu-3 lung adenocarcinoma cell line. RT-qPCR of RNA extracted from ESG cultures and Calu-3 cells. CFTR measured by Taqman assay and normalized to β2 microglobulin. Protocols and primers are shown in the Supplementary methods.

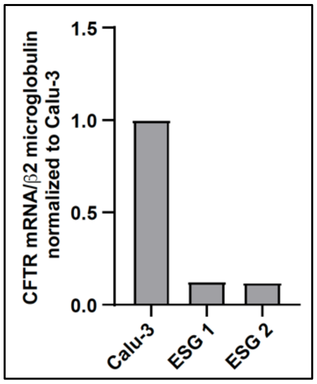

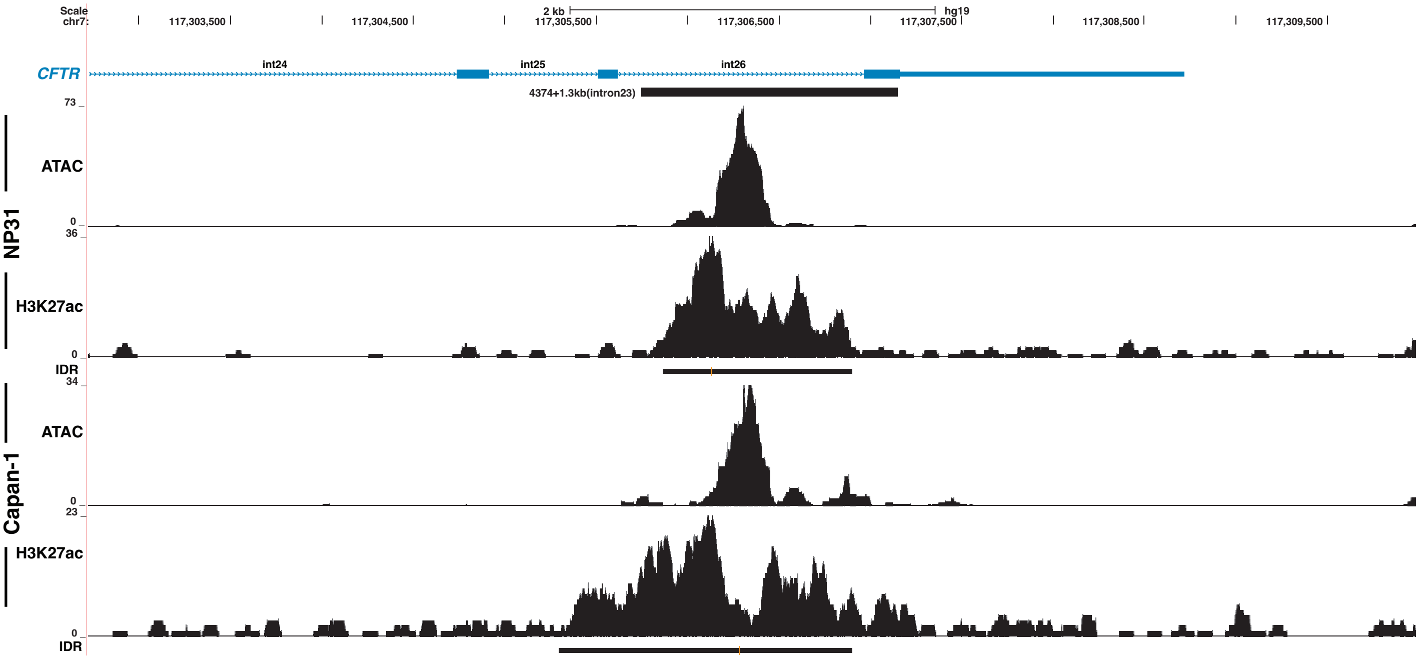

A

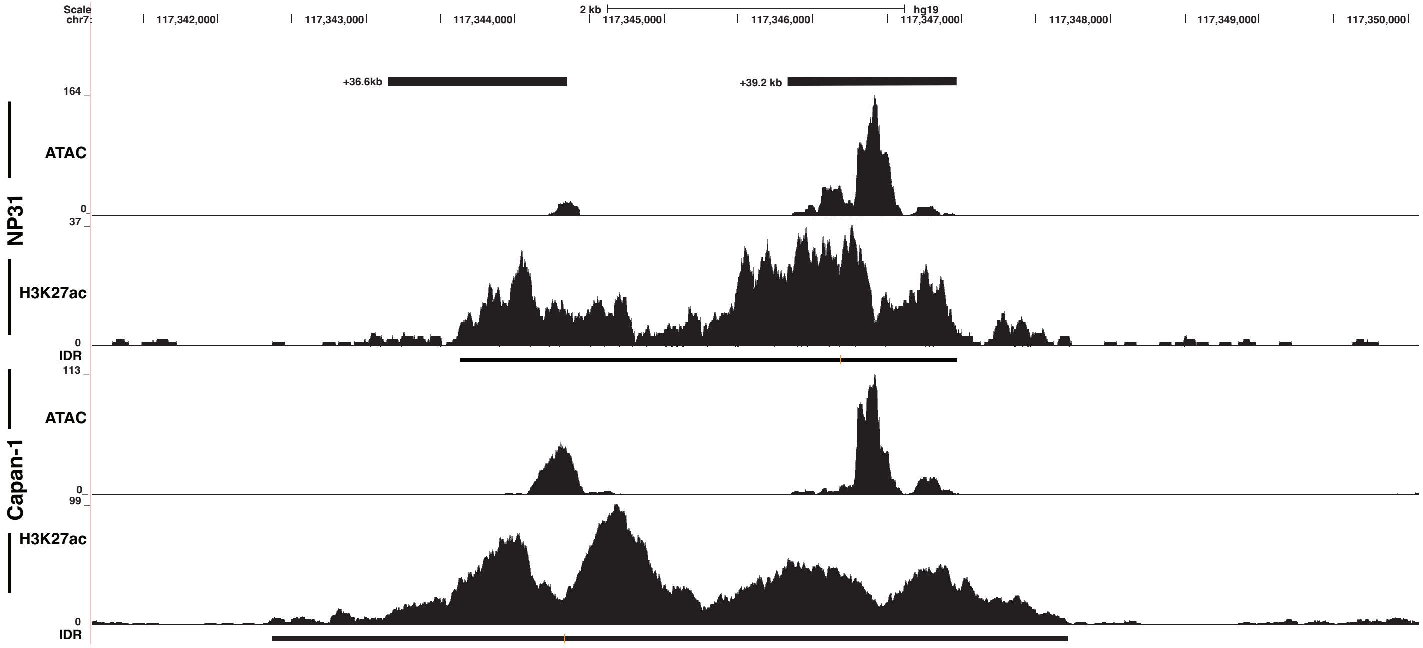

B

Figure S3. Open chromatin and histone modifications in NP31 and Capan-1 cells. Enlarged image of data shown in Fig.3A and 3B focusing on : **A**. The intron 23 DHS region; **B**. The +36.6 kb and +39.2 kb regions. Data are described fully in the legend to Fig. 2.

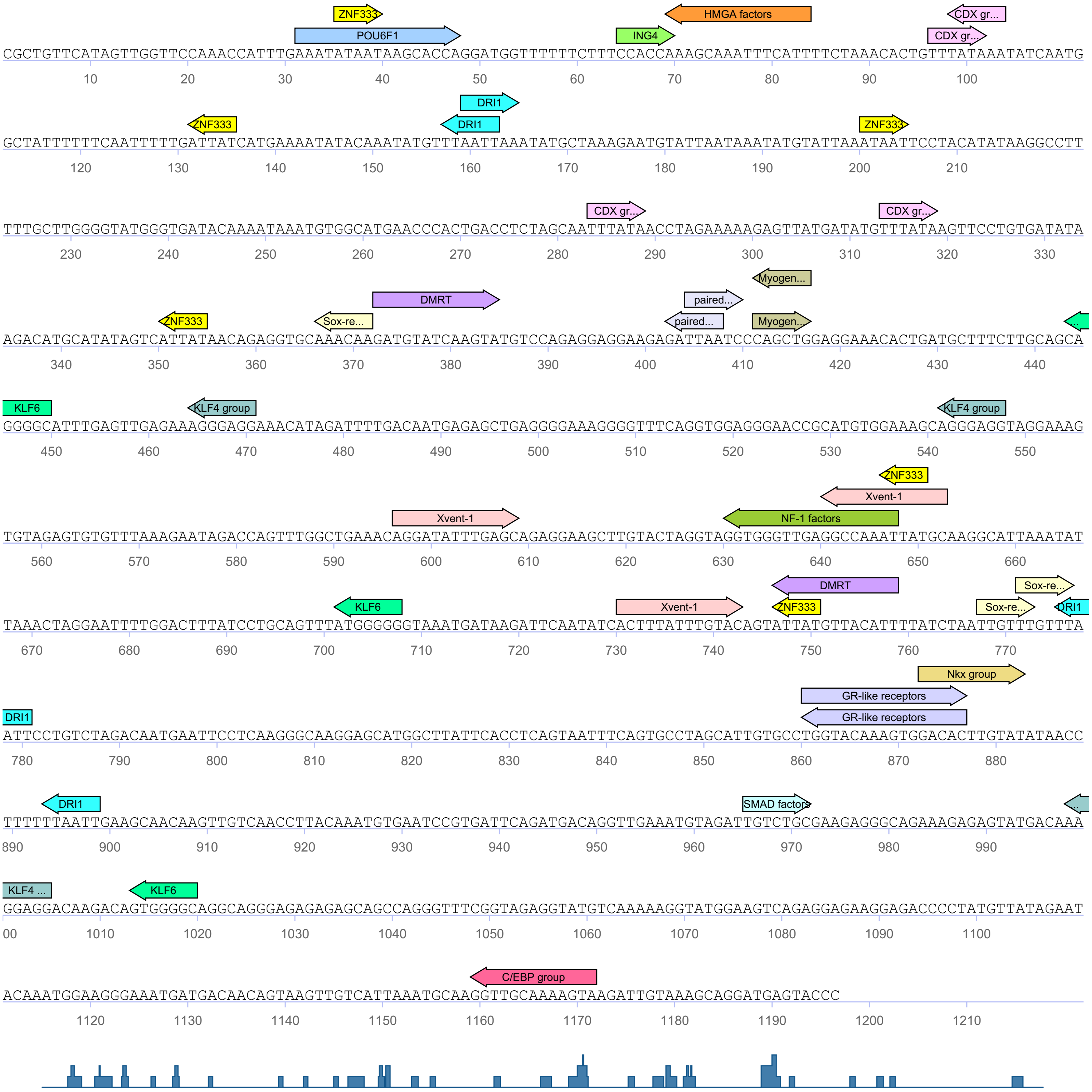

Figure S4. Predicted transcription factor binding sites in the intron 15 open chromatin peak from Transfac 2.0.

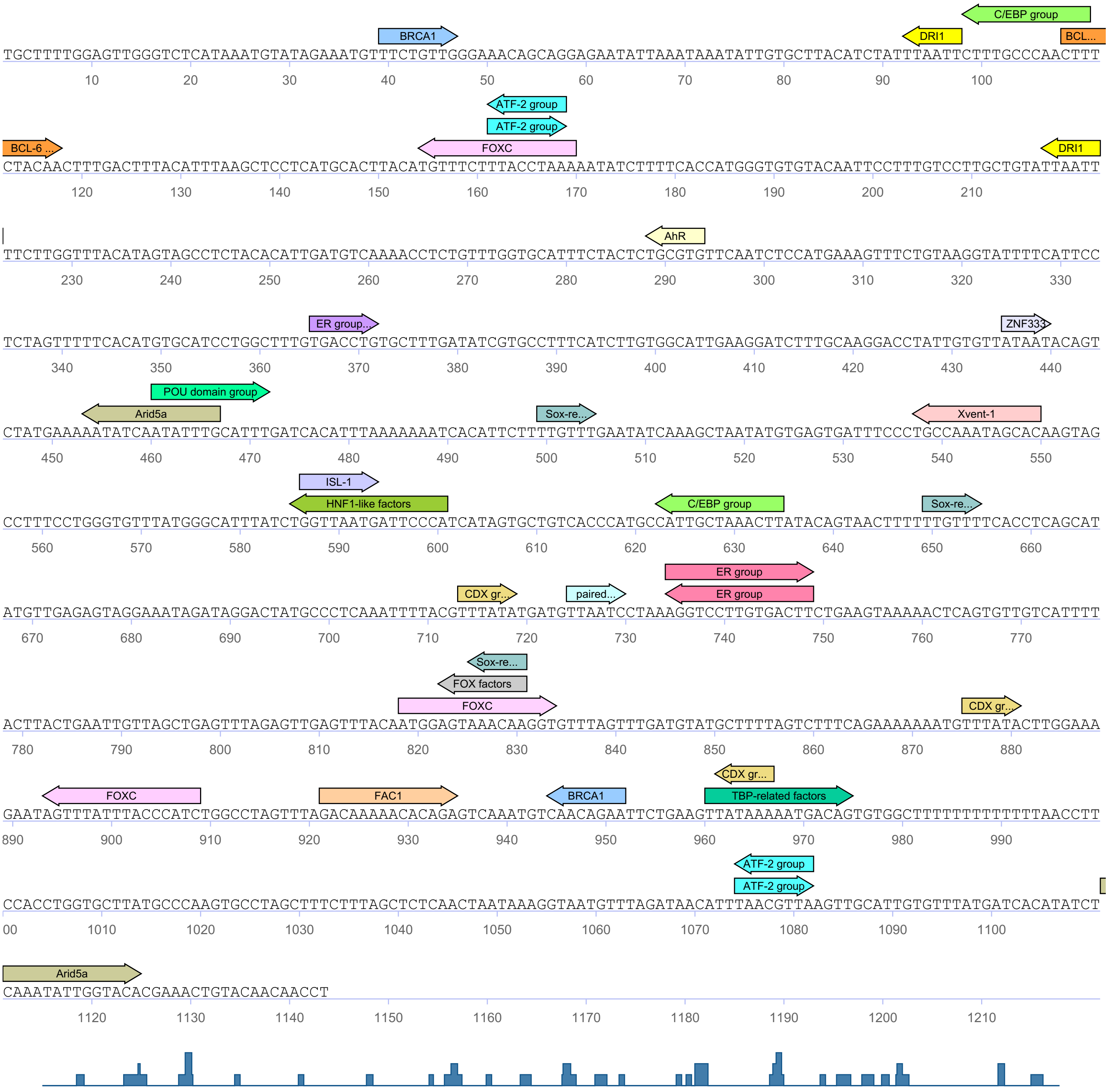

Figure S5. Predicted transcription factor binding sites in the intron 18 open chromatin peak from Transfac 2.0.

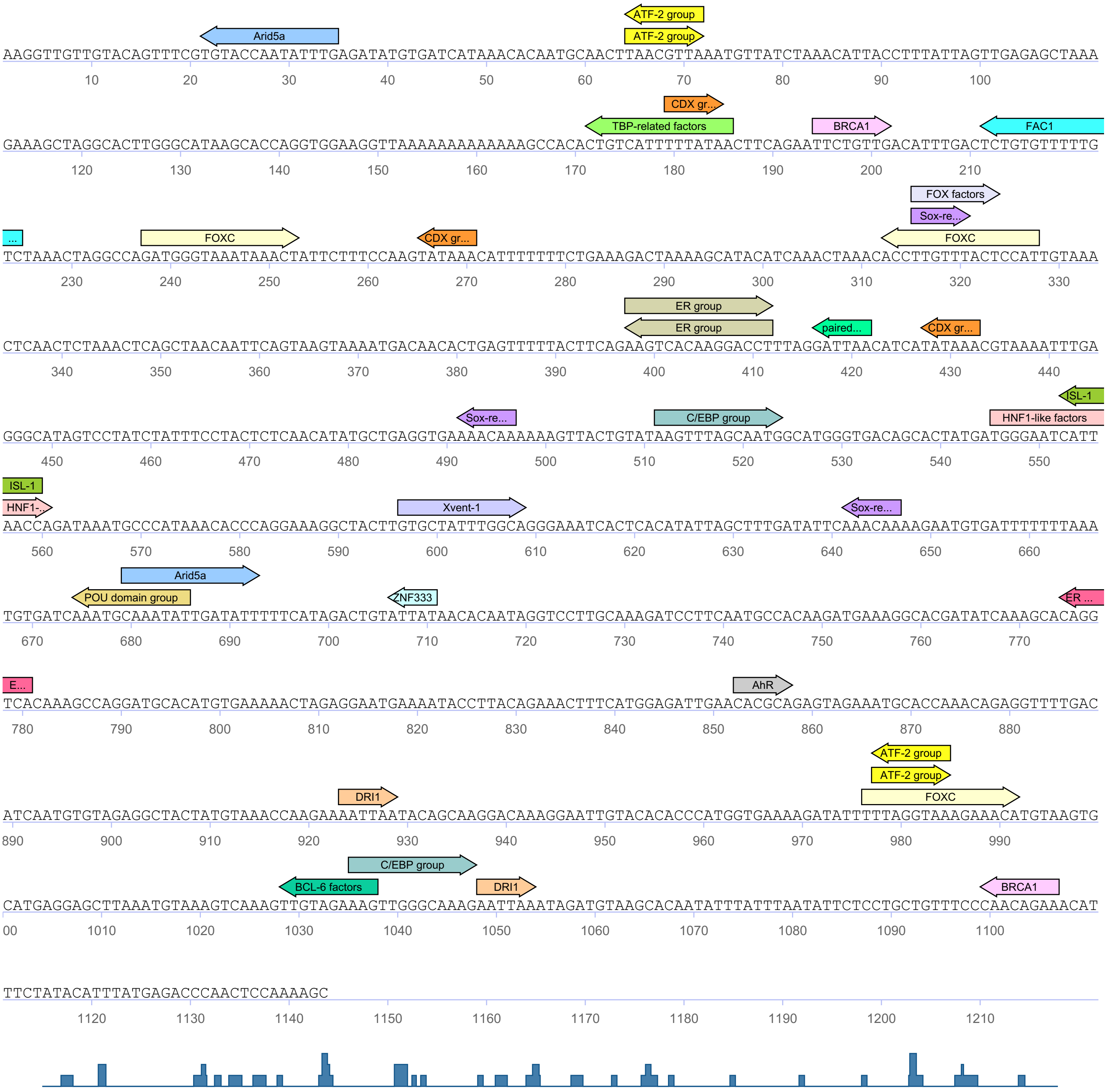

Figure S6. Predicted transcription factor binding sites in the intron 20 open chromatin peak from Transfac 2.0.

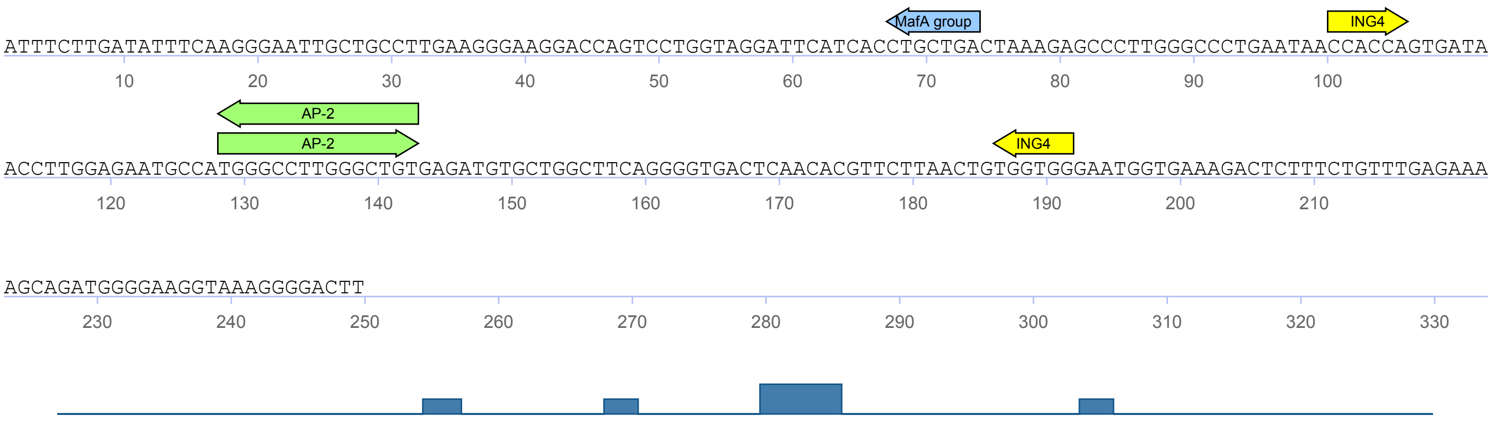

-33 kb Peak 1

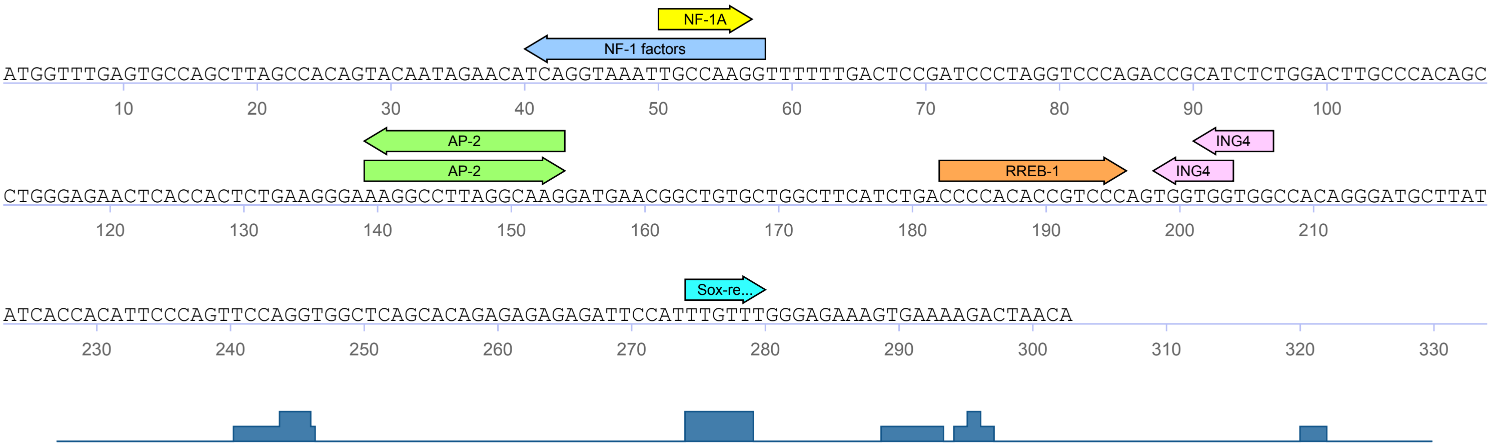

-33kb Peak 2

Figure S7. Predicted transcription factor binding sites in the -33 kb open chromatin peak from Transfac 2.0.

DNA sequence at core of -31 kb Peak of Open Chromatin

5'AGAAGAGGAGCTTGCTGACCTGAAGGAGAAGTCCCAGACCTGGCAACATTCACCGCAAGCTGGGTGGAAGAGTCC
TTGGGCTTTAAACATCACGGTGGCC3'

Predicted transcription factor binding sites by AliBaba2.1

AliBaba2.1 predicts the following sites in your sequence

```
Sequence seq_257

==================================================================================
seq(    0..   59)     agaagaggagcttgctgacctgaaggagaagtcccagacctggcaacattcaccgcaagc
Segments:
1.1.1.1    9    18             ===c-Jun==
2.1.1.4    13   22                 =====ER===
9.9.853    14   23                  =T3R-beta1
4.1.1.0    25   34                             ===c-Rel==
9.9.590    25   34                             =NF-kappaB
2.1.2.2    32   41                                    =RXR-alpha
==================================================================================
seq(   60..  119)     tgggtggaagagtccttgggctttaaacatcacggtggcc
Segments:
2.3.1.0    61   70     ====Sp1===
3.1.1.12   83   92                           ===HNF-1==
1.1.1.6    84   93                            ==CRE-BP1=
1.1.2.0    84   93                            ====CREB==
2.3.3.0    84   93                            =CPE_bind=
9.9.561    90   99                                  ==NF-muE1=

12 segments in this sequence identified as potential binding sites
```

Figure S8. Predicted transcription factor binding sites in the -31 kb open chromatin peak from AliBaba2.1.
