# Supplementary material for: CFTR Gene Regulation in Human Pancreatic Duct, Bile Duct and Sweat Gland Epithelial Cells
Source: J Cell Mol Med. 2025 Aug 10;29(15):e70751. doi: 10.1111/jcmm.70751 (PMC12336291; doi:10.1111/jcmm.70751)
Supplement: Supplementary file 2 — Table S1–S2: jcmm70751‐sup‐0002‐TableS1‐S2.docx. [file JCMM-29-e70751-s001.docx]

| **ChIP-qPCR primer sets** | **Sequences (5’>3’)** |
| --- | --- |
| CFTR -44kb F | AGTGAGATTAGTTGTCTCTTTTGGAGATAA |
| CFTR -44kb R | CCCTTGACTATTTTGTGCACATG |
| CFTR -35kb F | ATCTACCTTACCCTGCTGTCCATT |
| CFTR -35kb R | TCTGAATTATCAGCCCACAGTCA |
| CFTR -2kb Promoter F | TTGAACAATTTTCTGGTGGATAAGTC |
| CFTR -2kb Promoter R | ATGCACTAATTGCGACATGATATTC |
| CFTR -0.5kb Promoter F | GTTCTCCCGCCGGTGG |
| CFTR -0.5kb -0.5kb R | CAGTCGCGGCCTCTCTTTAG |
| CFTR DHS1 F | TCATTGTCAACTGTCAGGTAGCAA |
| CFTR DHS1 R | CAGAGTTAGGATTCCAGCCAGG |
| CFTR DHS10a,b F | TGCTTTATTGAATGGCATTACCTCTA |
| CFTR DHS10a,b R | AGATGCTTGTGGTAAGGGAGGAG |
| CFTR DHS11 F | TCCAAAAGCTGAGACAGGAAACT |
| CFTR DHS11 R | ATTACATACACACAAAAGTACACACATGACT |
| CFTR int15 F | TATAAGTTCCCCGTGGTTCC |
| CFTR int15 R | AATGAGGAGCTTGGAAAAGA |
| CFTR int18a F | GATCAACTCCTGTCAGAACAAACA |
| CFTR int18a R | AGACACATGAACCAGAAGAGAGC |
| CFTR int20 F | CTTCATAGCTGGAGAGTTTCCTAGC |
| CFTR int20 R | GCTGCTGATGGCATGGG |
| CFTR +15.6 F | ATCCATTTTCTTCAAGTCTCTCTCCAT |
| CFTR +15.6 R | GGAATGAGGATTGTTTATGATTTGG |
| WNT2 Promoter F | GGGAGAAACGCGGGGTTTTA |
| WNT2 Promoter R | TCGTAACCATGCAGCGATGT |

**Supplementary Table 1** Primer sequences used for ChIP-qPCR of histone modifications.

| **Viewpoint** | **Primary enzyme** | **Secondary enzyme** | **Reading primer (5’>3’)** | **Non-reading primer (5’>3’)** |
| --- | --- | --- | --- | --- |
| CFTR promoter  No barcode | NlaIII | Csp6I | AATGATACGGCGACCACCGAA  CACTCTTTCCCTACACGACGCT  CTTCCGATCTGCACTTACTAT  ATGCAGGCATG | CAAGCAGAAGACGG  CATACGATGAAGTG  TTCTTTGGATATTG  C |
| CFTR promoter  CG | NlaIII | Csp6I | AATGATACGGCGACCACCGAA  CACTCTTTCCCTACACGACGCT  CTTCCGATCTCGGCACTTACT  ATATGCAGGCATG | CAAGCAGAAGACGG  CATACGATGAAGTG  TTCTTTGGATATTG  C |
| -80.1 kb  No barcode | NlaIII | Csp6I | AATGATACGGCGACCACCGAA  CACTCTTTCCCTACACGACGCT  CTTCCGATCTACTGAGAACTT  ACAGGGCAGTC | CAAGCAGAAGACGG  CATACGACTGGTAG  CTTTTGGTTGAATG |
| -80.1 kb  CG | NlaIII | Csp6I | AATGATACGGCGACCACCGAA  CACTCTTTCCCTACACGACGCT  CTTCCGATCTCGACTGAGAAC  TTACAGGGCAGTC | CAAGCAGAAGACGG  CATACGACTGGTAG  CTTTTGGTTGAATG |

**Supplementary Table 2** Restriction enzyme pairs and primer sequences used for 4C library generation.
