# Supplementary material for: CFTR Gene Regulation in Human Pancreatic Duct, Bile Duct and Sweat Gland Epithelial Cells
Source: J Cell Mol Med. 2025 Aug 10;29(15):e70751. doi: 10.1111/jcmm.70751 (PMC12336291; doi:10.1111/jcmm.70751)
Supplement: Supplementary file 3 — Data S1: jcmm70751‐sup‐0003‐DataS1.docx. [file JCMM-29-e70751-s003.docx]

**Supplemental methods:**

**For cholangiocyte organoid RT-qPCR:**

RNA was extracted using the RNAqueous Micro Kit (Invitrogen, TM1931). RNA (1ug) was reverse transcribed into cDNA with iScript™ reverse transcriptase (BIO-R AD, 1708841). Quantitative PCR was then performed on the samples with SYBR-Green (BIO-RAD, 1725274) in the CFX96 Maestro machine (BIO-RAD). Expression levels were normalized to the housekeeping gene TATA box-binding protein (TBP). For control, total RNA samples for AL (adult human liver, Clontech, 636531) and PANC (adult human pancreas, Clontech, 636577) were used as reference controls. Oligonucleotide sequences are listed in the table below.

**For sweat gland duct cell RT-qPCR:**

Total RNA was extracted using TRIzol (Thermo Fisher, 15596018) according to the manufacturer's protocol. cDNA prepared using Taqman Reverse Transcription Reagents (Thermo Fisher, N8080234) with random hexamers. *CFTR* was assayed using the following primers and normalized to β2 microglobulin (B2M).

CFTR
TAQEX5F: AGCTGTCAAGCCGTGTTCTAGATA
TAQEX6AR: ATGAGGAGTGCCACTTGCAAA
/56-FAM/CACACGAAA/ZEN/TGTGCCAATGCAAGTCCTT/3IABkFQ/


B2M
B2M_TaqMan FWD: AAGTGGGATCGAGACATGTAAG
B2M_TaqMan REV: GCAAGCAAGCAGAATTTGGA
56-JOEN/TCATGGAGG/ZEN/TTTGAAGATGCCGCA/3IABkFQ
